# *S*. *pombe wtf* drivers use dual transcriptional regulation and selective protein exclusion from spores to cause meiotic drive

**DOI:** 10.1371/journal.pgen.1009847

**Published:** 2022-12-07

**Authors:** Nicole L. Nuckolls, Ananya Nidamangala Srinivasa, Anthony C. Mok, Rachel M. Helston, María Angélica Bravo Núñez, Jeffrey J. Lange, Todd J. Gallagher, Chris W. Seidel, Sarah E. Zanders

**Affiliations:** 1 Stowers Institute for Medical Research, Kansas City, Missouri, United States of America; 2 Department of Cell Biology and Physiology, University of Kansas Medical Center, Kansas City, Kansas, United States of America; 3 University of Missouri—Kansas City, Kansas City, Missouri, United States of America; The University of North Carolina at Chapel Hill, UNITED STATES

## Abstract

Meiotic drivers bias gametogenesis to ensure their transmission into more than half the offspring of a heterozygote. In *Schizosaccharomyces pombe*, *wtf* meiotic drivers destroy the meiotic products (spores) that do not inherit the driver from a heterozygote, thereby reducing fertility. *wtf* drivers encode both a Wtf^poison^ protein and a Wtf^antidote^ protein using alternative transcriptional start sites. Here, we analyze how the expression and localization of the Wtf proteins are regulated to achieve drive. We show that transcriptional timing and selective protein exclusion from developing spores ensure that all spores are exposed to Wtf4^poison^, but only the spores that inherit *wtf4* receive a dose of Wtf4^antidote^ sufficient for survival. In addition, we show that the Mei4 transcription factor, a master regulator of meiosis, controls the expression of the *wtf4^poison^* transcript. This transcriptional regulation, which includes the use of a critical meiotic transcription factor, likely complicates the universal suppression of *wtf* genes without concomitantly disrupting spore viability. We propose that these features contribute to the evolutionary success of the *wtf* drivers.

## Author summary

Genomes are often considered a collection of ‘good’ genes that provide beneficial functions for the organism. From this perspective, disease is thought to arise due to disfunction of ‘good’ genes. For example, infertility can be caused by the failure of a gene that normally helps fertility. This view is incomplete as ‘parasitic’ genes that provide no benefit to the organism also exist. These genes can also contribute to disease, often as a result of the mechanisms they use to ensure their transmission to the next generation. For example, killer meiotic drivers are found throughout eukaryotes and contribute to infertility by actively destroying the gametes (e.g., egg and sperm) that do not inherit them. In this work we study the transcriptional regulation of *wtf4*, a model killer meiotic driver found in fission yeast to understand mechanisms of drive. The *wtf4* gene encodes both a poison and an antidote protein on largely overlapping coding sequences. We found that different promoters and differential localization properties of the poison and antidote proteins both facilitate killer meiotic drive. We also found that the expression of the poison protein relies on a key transcription factor essential for gametogenesis. The use of this transcription factor likely complicates suppressing *wtf4* without compromising gametogenesis. This feature likely contributes to the evolutionary success of the *wtf* drivers, which are found in many copies in fission yeast genomes.

## Introduction

The transmission of most eukaryotic genes follows Mendel’s first law of segregation. This law stipulates that the two alleles of a heterozygous organism (e.g., A/a) segregate randomly into gametes such that each allele is transmitted to 50% of the progeny [1]. There are, however, alleles that can break Mendel’s law to force their own transmission into more than half of the offspring. These lawbreaking genes are called meiotic drivers [2,3]. There is a tremendous diversity of meiotic drive genes with distinct evolutionary origins and mechanisms found throughout eukaryotes [4–28]. However, the molecular details underlying how these systems are expressed and function are limited. Uncovering these details is important for understanding meiotic drive and, more broadly, has the potential to reveal novel insights about gametogenesis. For example, the existence of a sperm-autonomous phenotype, despite cytoplasmic connections between sister sperm, was discovered through study of the *t*-haplotype driver in mouse [14].

Meiotic drivers can generally be considered selfish or parasitic genes [29]. This is because drivers can persist in genomes due to the transmission advantages of drive, rather than due to fitness benefits they provide to the organisms that carry them. In fact, meiotic drivers often cause decreased fitness through a variety of direct and indirect mechanisms [30–33]. The fitness costs are especially deleterious amongst the class of drivers known as the killer meiotic drivers (reviewed in [34]). These drivers can achieve up to 100% transmission to viable gametes by destroying the products of meiosis that do not inherit the driver from a heterozygote.

**Fig 1:**
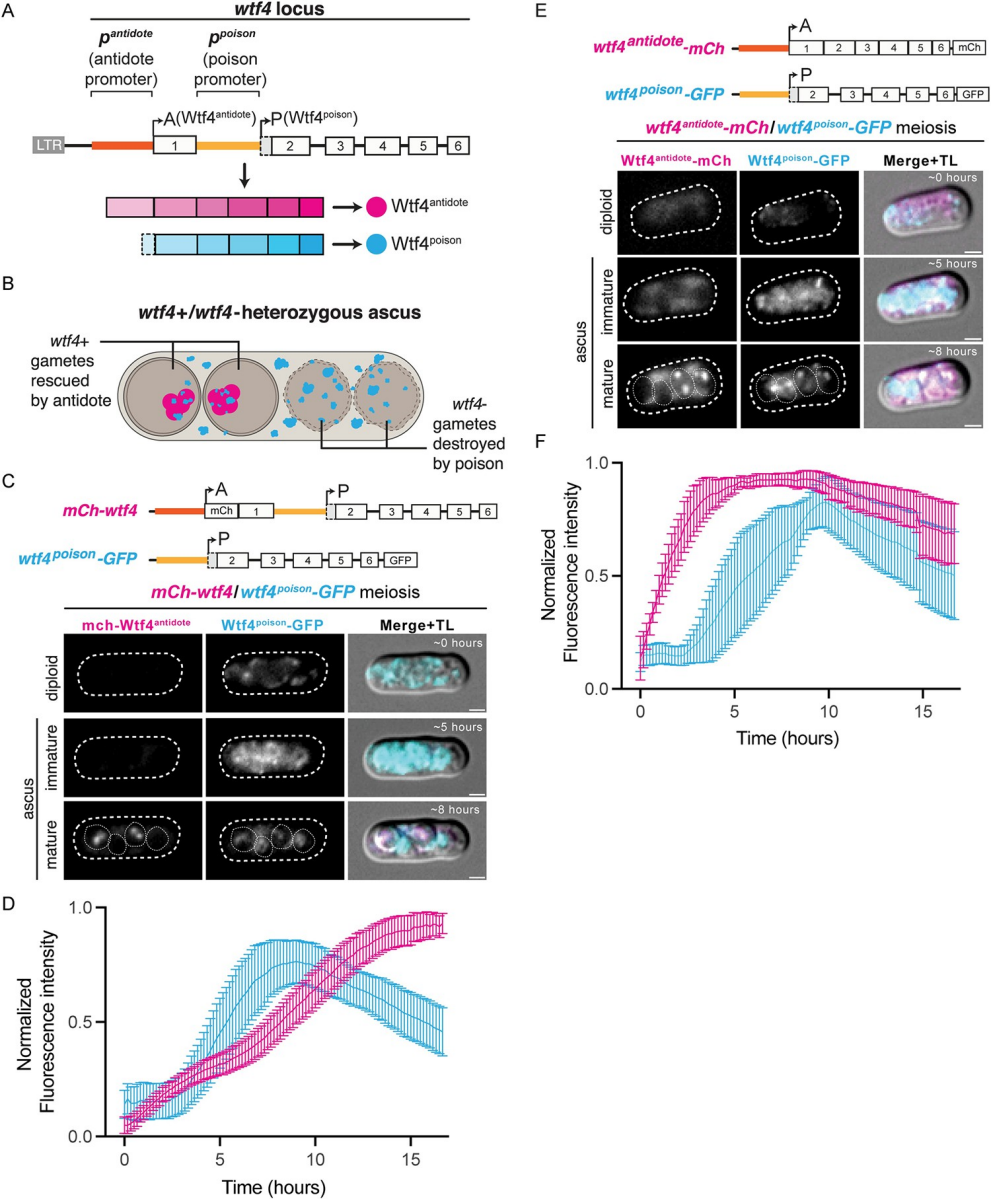
Wtf4^poison^ and Wtf4^antidote^ proteins have distinct expression profiles. (**A**) A depiction of the *wtf4* gene is shown. There is a Tf retrotransposon LTR located 284 base pairs upstream of exon 1. The sequence upstream of exon 1 contains the antidote promoter, *p*^*antidote*^ (in orange) and intron 1 contains the poison promoter, *p*^*poison*^ (in yellow). The arrows represent the predicted translational start sites. Wtf4^antidote^ (magenta circle) is encoded by exons 1–6. Wtf4^poison^ (cyan circle) is encoded by exons 2–6. (**B**) Model of Wtf4 poison-antidote meiotic drive from *wtf4* heterozygotes. Wtf4^poison^ is present in all four spores, while Wtf4^antidote^ expression is enriched in only two of the four spores. (**C**) Time-lapse microscopy of an *mCherry-wtf4/wtf4*^*poison*^*-GFP* diploid undergoing meiosis and sporulation. mCherry-Wtf4 is shown in magenta and Wtf4^poison^-GFP is shown in cyan in merged images. Images of a representative cell from the time lapse shown at ~ 0 hours (diploid), ~5 hours (immature ascus), and ~8 hours (mature ascus) after the video begins. Distinct images for these strains were first presented in [37] and a timelapse following protein movement in mature asci of these strains was presented in [52]. (**D**) Normalized fluorescence intensity plots of Wtf4^poison^-GFP (cyan line) and mCherry-Wtf4^antidote^ (magenta line) over the course of the time lapse (n = 25). (**E**) Time-lapse microscopy of a *wtf4*^*antidote*^*-mCherry/wtf4*^*poison*^*-GFP* diploid undergoing meiosis and sporulation. Wtf4^antidote^-mCherry is shown in magenta and Wtf4^poison^-GFP is shown in cyan in merged images. Images of a representative cell from the time lapse shown at ~ 0 hours (diploid), ~5 hours (immature ascus), and ~8 hours (mature ascus) after the video begins. (**F**) Normalized fluorescence intensity plots of Wtf4^poison^-GFP (cyan line) and Wtf4^antidote^-mCherry (magenta line) over the course of the time lapse (n = 13) TL = transmitted light. All scale bars represent 2 μm. Error bars depict the 95% confidence interval. Time lapse imaging was performed under the same settings. Not all images are shown at the same brightness and contrast to avoid over saturation of pixels in the brighter images.

Despite the fitness costs of killer meiotic drivers, many populations harbor these genes (reviewed in [34]). However, the fission yeast *S*. *pombe* may represent an extreme case. Different natural isolates of *S*. *pombe* contain between 4–14 predicted killer meiotic drivers from the *wtf* (with transposon fission yeast) gene family [35–38]. These genes kill the meiotic products (spores) that do not inherit them from a heterozygote using two proteins produced from two transcripts with largely overlapping coding sequences: a Wtf^poison^ protein that kills spores and a Wtf^antidote^ protein that rescues spores that inherit the driver (Fig 1A and 1B; [37,38]). After meiosis in *wtf* driver heterozygotes, the Wtf^poison^ is found in all spores, while the Wtf^antidote^ is enriched in those that inherit the *wtf* driver [37,39]. These distribution patterns explain how the driver has the potential to kill all spores, yet specifically rescues those that inherit the driver (Fig 1B). How this dual Wtf protein localization pattern is established and maintained is unclear.

Uncovering the mechanisms of meiotic drivers is important for understanding how these parasites impact the organisms that carry them. The *wtf* genes, in particular, confer no known fitness benefits and are largely responsible for the infertility observed in heterozygous *S*. *pombe* diploids generated by outcrossing between distinct isolates [40–45]. Due to the fitness costs of *wtf* drivers, *S*. *pombe* has likely evolved ways to tolerate or suppress drive. There are currently two described mechanisms that can suppress *wtf* meiotic drivers: suppression by other *wtf* genes and gamete disomy [39–41]. Transcriptional silencing operating through trans-acting regulators or cis-acting chromatin packaging are additional candidates for suppression mechanisms. While this possibility has not been explored during spore formation, histone deacetylation and TOR-mediated RNA processing have been shown to contribute to mitotic silencing of *wtf* genes [46–49].

Our goals in this study were to understand how the expression and localization of Wtf driver proteins are coordinated and to find possible routes to transcriptional suppression of *wtf* drivers. To do this, we use the *wtf4* gene from the *S*. *kambucha* natural isolate of *S*. *pombe* as a model system. We define the promoter sequences that control the *wtf4^poison^* and *wtf4^antidote^* transcripts. We demonstrate that differential expression due to the distinct promoters and differential inclusion of the two Wtf4 proteins in developing spores both contribute to Wtf4 protein localization and thus efficient drive. We also found that the expression of the *wtf4^poison^* transcript is controlled by the transcription factor Mei4, a master regulator of gene expression in meiosis [50,51]. In the context of our results, we discuss why evolving or maintaining transcriptional silencing of *wtf* genes and drivers in general may be challenging.

## Results

### Wtf4^poison^ and Wtf4^antidote^ proteins have distinct expression profiles

In previous work, we used fluorescent markers to separately tag and visualize the Wtf4 proteins [37,52]. We imaged mostly mature asci (sacks holding spores) and diploid cells present at the same time points that had not completed spore formation. In diploid cells, these analyses revealed signal from a tagged Wtf4^poison^-GFP allele, but not an mCherry-Wtf4^antidote^ allele. In mature asci, we observed Wtf4^poison^-GFP in all spores and mCherry-Wtf4^antidote^ strongly enriched in spores that inherited the locus encoding the allele [37,52]. These analyses, however, were too coarse to reconstruct the full expression dynamics of the two proteins.

In this work, we explored the expression of the two Wtf4 proteins more thoroughly to uncover how the two proteins achieve targeted destruction of spores that do not inherit *wtf4* from a heterozygote (Fig 1B). We again used *wtf4^poison^-GFP*, a separation-of-function allele that does not express Wtf4^antidote^ and adds a GFP tag to the C-terminus of the Wtf4^poison^ (S1 Fig allele 1). We have previously shown that this allele acts as a functional poison but is slightly less toxic than the corresponding untagged *wtf4^poison^* allele ([37], S2A Fig, diploid 4). We also used the *mCherry-wtf4* allele, which tags Wtf4^antidote^, but still generates an untagged Wtf4^poison^ (S1 Fig allele 2). This allele functions similarly to the wild type *wtf4* in allele transmission and viable spore yield (VSY) assays ([37], S2A Fig, diploid 3). Viable spore yield is a measure of fertility that assays the number of viable spores generated per cell induced to form spores [53]. In this work, we normalized viable spore yields to those measured in wild-type control cells with an empty vector integrated in the genome instead of a *wtf4* allele. Those wild-type cells are therefore considered to have 100% fertility. Complete drive in a heterozygote is expected to reduce fertility by roughly 50% and expression of a Wtf4^poison^ allele in the absence of Wtf4^antidote^ is expected to reduce fertility more than 50%.

We integrated the *mCherry-wtf4* and *wtf4^poison^-GFP* alleles mentioned above into the *ade6* locus of different haploid strains. We crossed these two strains together to generate heterozygous *mCherry-wtf4 / wtf4^poison^-GFP* diploids. We then completed time-lapse microscopy during fission yeast meiosis and spore formation (Fig 1C and 1D). In this text, we depict Wtf4^antidote^ in magenta and Wtf4^poison^ in cyan in all the images, regardless of fluorescent protein tag. We consistently observed Wtf4^poison^-GFP hours before mCherry-Wtf4^antidote^ (Fig 1C and 1D). Given that GFP maturation is only minutes faster than mCherry [54–56], the earlier signal from Wtf4^poison^-GFP cannot be explained by maturation times of the fluorescent proteins alone. Through the time-course, the GFP signal decreases, but then increases to reach high intensity prior to spore formation. The signal was found in all four spores, not just those that inherited the *wtf4^poison^-GFP* allele (Fig 1C and 1D, S1 Video panel A). These observations suggested either that most of the Wtf4^poison^ protein is produced prior to spore individualization or that the *wtf4^poison^* transcript and/or protein is freely exchanged between spores. We distinguished between these models by photobleaching two spores or the entire ascus in asci expressing Wtf4^poison^-GFP and assaying recovery of fluorescence (i.e., FRAP). We found minimal recovery of Wtf4^poison^-GFP signal after photobleaching both spores and asci (S3A–S3D Fig). These data support that most Wtf4^poison^ is produced prior to spore formation and then packaged within all spores.

The mCherry-Wtf4^antidote^ signal was very low prior to spore formation and reached max intensity after spore formation (Fig 1C and 1D, S1 Video panel A). We were curious if the transcription of the *wtf4^poison^* could interfere with the transcription of the *wtf4^antidote^* prior to spore formation because the *wtf4^poison^* transcriptional start site is downstream of the *wtf4^antidote^* transcriptional start site. To test this, we generated an alternate allele of *mCherry-wtf4^antidote^* that lacks introns, and thus lacks the capacity to produce a poison transcript (Figs 1A and S1 allele 3). This allele encodes a functional antidote as it suppresses drive of wild type *wtf4*, but does not cause drive alone (S2A Fig, diploids 10 and 11). In heterozygotes with this allele integrated at *ade6*, we again saw minimal signal from this mCherry-Wtf4^antidote^ protein in diploids and strong enrichment in two spores. This suggests *wtf4^poison^* expression is not significantly affecting the gross localization dynamics of mCherry-Wtf4^antidote^ (S2B Fig).

We also tested if the difference in signal we observed between the tagged Wtf4^antidote^ and Wtf4^poison^ proteins could be due to differences in the N- versus C-terminal tags. To test this, we assayed a C-terminally tagged Wtf4^antidote^ separation-of-function allele, *wtf4^antidote^-mCherry*. This allele does not encode Wtf4^poison^ because it lacks introns (Figs 1A and S1 allele 4). We integrated this allele into the *ura4* locus and found that it indeed acted as an antidote-only allele because it does not drive on its own, but it does suppress drive of *wtf4* (S2A Fig, diploids 8 and 9). We then crossed a strain carrying the *wtf4^antidote^-mCherry* allele to haploid cells carrying *wtf4^poison^-GFP* to generate *wtf4^antidote^-mCherry/ura4+, wtf4^poison^-GFP/ade6+* diploids. We imaged these diploids over a time course and observed that the Wtf4^antidote^ localization was different than the pattern we observed with the *mCherry-wtf4* allele in that the cells had mCherry signal prior to spore formation (Fig 1E and 1F, S1 Video panel B). Specifically, we saw Wtf4^antidote^-mCherry in diploid cells induced to undergo meiosis and in the ascal cytoplasm (i.e., outside of spores) of immature asci (Figs 1E and 1F and S2C, S1 Video panel B). In mature asci, Wtf4^antidote^-mCherry was strongly enriched in two of the four spores, similar to the N-terminally tagged protein, but significant signal remained in the ascal cytoplasm outside spores Figs 1E and 1F and S2C, S1 Video panel B). Another C-terminally tagged Wtf4^antidote^ separation-of-function allele, *wtf4^antidote^-GFP* (S1 Fig allele 5), localized similarly to *wtf4^antidote^-mCherry* (S2C and S2D Fig) and behaved as a functional antidote allele (S2A Fig, diploids 13 and 15).

We assayed expression of the Wtf4 proteins via western blots. In samples from *wtf4^antidote^-mCherry/ura4+, wtf4^poison^-GFP/ade6+* diploids, we detected free GFP and a band we infer to be full-length Wtf4^poison^-GFP when cells are undergoing meiosis and when mature spores are present. We never detect full-length Wtf4^antidote^-mCherry, but we detect free mCherry when cells are undergoing meiosis and when mature spores are present (S4 Fig). In diploids expressing the *mcherry-wtf4^antidote^* allele, we also only detect free mCherry, but only when mature spores are present, consistent with our image analyses showing mCherry only in spores (S5 Fig). Our inability to detect full-length tagged Wtf4^antidote^ on a western was not surprising as we previously showed the protein is quickly trafficked to vacuoles, where it is presumably degraded [52].

We also attempted to detect Wtf4^poison^-GFP and Wtf4^antidote^-mCherry on Western blots from samples collected from cells induced to undergo synchronous meiosis using an analog sensitive allele of the Pat1 kinase that inhibits meiotic entry (*pat1*.*L95G*; [57]). The results were similar to our results in wild-type cells, described above, except we could detect a faint band early in meiosis that may be Wtf4^antidote^-mCherry, in addition to the free mCherry band observed at all timepoints (S6 Fig). Overall, the westerns support our imaging as we see signal from the tagged Wtf4^poison^ locus when cells are undergoing meiosis and in samples containing spores. We observe signal from both tagged Wtf4^antidote^ alleles in samples containing spores, but only from the C-terminally tagged allele in samples collected prior to spore formation (S5 and S6 Figs).

We conclude that the C-terminally tagged alleles reveal a Wtf4^antidote^ protein population present prior to spore formation that is not apparent with the N-terminally tagged alleles. The nature of the additional protein is not clear. The early Wtf4^antidote^ protein could be produced using the second translational start site found in exon 1 (codon 12) via leaky transcriptional scanning [58]. This second start site can be used to encode a fully functional antidote protein (S1 Fig allele 6, S2A Fig diploids 6–7, [37]). In the two N-terminally Wtf4^antidote^ tagged alleles discussed above, the putative shorter Wtf4^antidote^ protein would not be tagged, even if it was produced. It is also possible the Wtf4^antidote^ population revealed with the C-terminal tag is full-length protein, but its expression prior to spore formation is disrupted by the N-terminal tag. We did not distinguish between these possibilities.

Overall, our results show that signal from the N-terminally tagged Wtf4^antidote^ used in our previous studies does not reflect the total Wtf4^antidote^ population. The C-terminally tagged alleles described here reveal additional Wtf4^antidote^ protein is present prior to spore formation. This interpretation is also consistent with previous long-read RNA sequencing data showing at least some transcription of *wtf* antidotes prior to spore formation (e.g., 0–6 hours after meiotic induction) [37,38,59]. Both alleles show that additional Wtf4^antidote^ protein production occurs after spore formation. In contrast, our time course analysis of Wtf4^poison^-GFP production revealed that most of the Wtf4^poison^ protein is present prior to spore formation and the protein cannot move freely between spores.

### Distinct promoters contribute to the distinct localization patterns of the Wtf4 proteins

**Fig 2:**
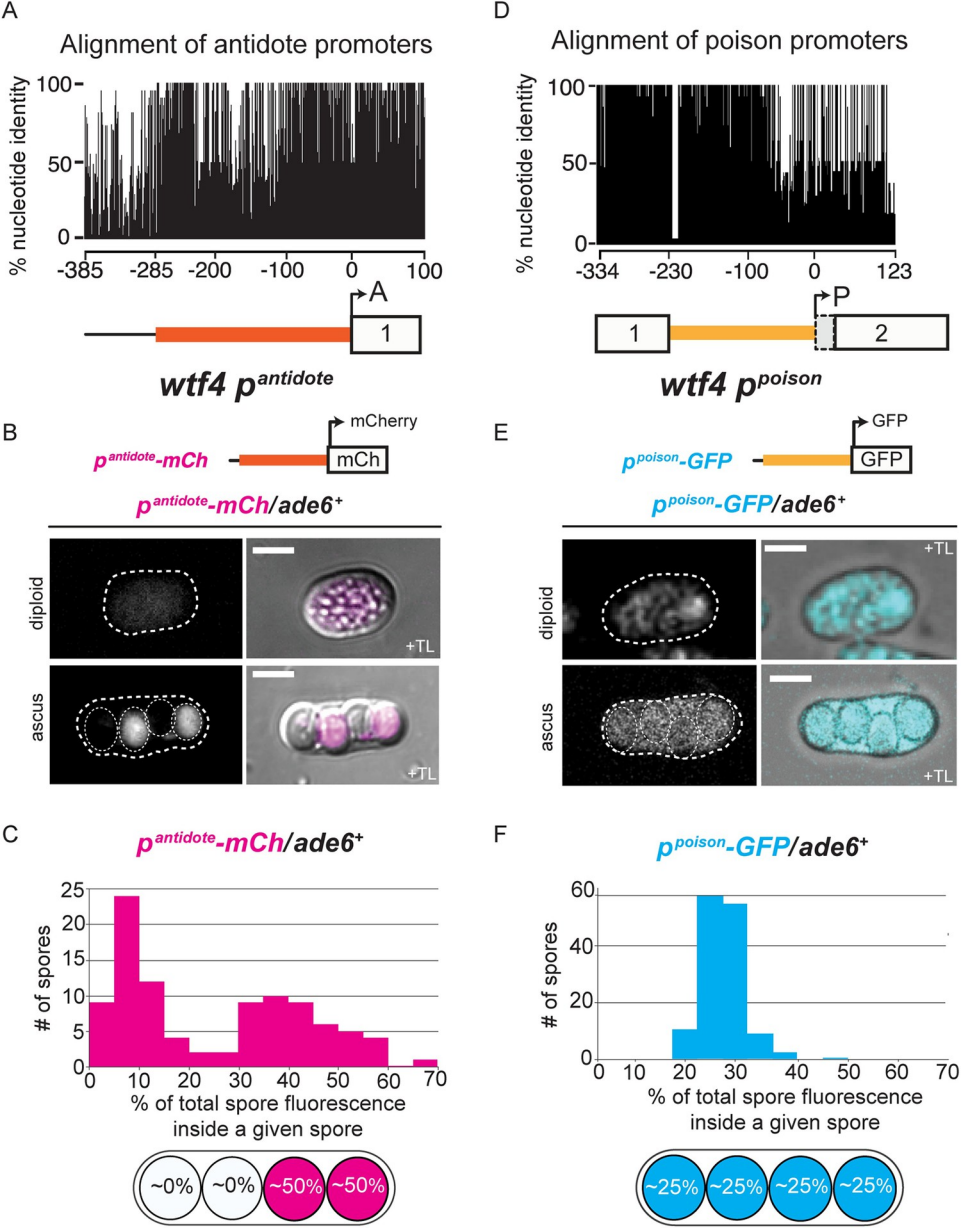
Distinct promoters largely explain differential localization of Wtf4^poison^ and Wtf4^antidote^ proteins. Depictions and alignments of (**A**) the *wtf4* antidote promoter and (**D**) the *wtf4* poison promoter are shown. For the alignment of antidote promoters, we aligned the 600 base-pairs upstream of 41 predicted antidote-only alleles [36] from three different strains of *S*. *pombe* (the reference genome, *S*. *kambucha*, and *FY29033*, [85]). For the alignment of poison promoters, we aligned the intron 1 sequences (flanked by sequences 100 base-pairs upstream and downstream of intron 1) of 28 predicted poison-antidote *wtf* drivers [36] from three different strains of *S*. *pombe* (the reference genome, *S*. *kambucha*, and *FY29033*). The image shows the percent identity at each nucleotide position, excluding gaps. (**B**) Images of *p*^*antidote*^-*mCherry/ade6+* diploid and ascus. (**C**) Quantification of mCherry fluorescence within *p*^*antidote*^-*mCherry/ade6+* asci (n = 24). (**E**) Images of *p*^*poison*^-*GFP/ade6+* diploid and ascus. (**F**) Quantification of GFP fluorescence within *p*^*poison*^-*GFP/ade6+* asci (n = 34). All images were acquired after 3 days on sporulation media. For quantification, we assayed the fluorescence intensity within each spore and then divided that number by the total fluorescence intensity within all 4 spores. TL = transmitted light. All scale bars represent 2 μm. Images of diploids are shown at the same brightness and contrast and were imaged at the same settings as the ascus generated from diploids of the same genotype.

We next wanted to explore if the distinct promoters contribute to the different localization patterns of the Wtf4^poison^ and Wtf4^antidote^ proteins in asci. To test this, we generated constructs with fluorescent proteins, mCherry and GFP, under the control of the *wtf4^antidote^* and *wtf4^poison^* promoters (including the 5’ untranslated regions (UTRs)), respectively. For the *wtf4^antidote^* promoter (*p^antidote^*), we used the 285 base pairs found upstream of exon 1. This is just downstream of a nearby Tf transposon long terminal repeat (LTR) that is not necessary for Wtf^antidote^ production (Fig 1A; [38,52]). Previous work assaying *cw9* and *cw27* (two *wtf* drivers in the CBS5557 isolate) found that the 288 bp upstream of exon 1 was insufficient to generate a fully functional *p^antidote^* promoter [38]. Although the promoter regions of the genes are highly similar, our previous results showed that for *wtf4*, 285 bp upstream sequence was sufficient to promote production of a functional Wtf4^antidote^ [52]. This sequence is also well conserved amongst *wtf* genes that encode for an antidote, which also supports that this region includes the promoter of *wtf4^antidote^*([36–38,59,60]; Fig 2A). To characterize the poison promoter (*p^poison^*), we used the 230 bp sequence that makes up intron 1 of the antidote transcript (Fig 2D). This sequence includes the transcriptional start site of the *wtf4^poison^* transcript and is well conserved amongst *wtf* drivers (Fig 2D, [36–38,59]).

We integrated the promoter reporter constructs at the *ade6* locus of different haploid strains. We then generated diploid cells heterozygous for each of the reporters individually (i.e., *reporter*/*ade6*+) and imaged them through meiotic induction and spore formation. With the *p^antidote^-mCherry* reporter, we observed low signal in diploids undergoing meiosis, but the strongest mCherry signal was observed in two out of the four spores (Fig 2B and 2C), presumably the two that inherited the reporter construct. Importantly, the observed localization of the *p^antidote^-mCherry* reporter protein supports that expression occurs both before and after spore individualization.

As mentioned above, a fully functional Wtf4^antidote^ protein can be made using a second ATG codon at the 12^th^ codon position [37]. We speculated that additional transcriptional regulatory sequences may be found in those coding sequences upstream of codon 12. To test this, we generated *p^antidote long^-mCherry*, a construct with mCherry under a *wtf4^antidote^* promoter that also contains the first 11 codons of the *wtf4^antidote^* coding sequence (S7B Fig). We integrated this allele at *lys4* and crossed this strain to wild type to generate heterozygous diploids. In these diploids and in the asci generated via these diploids, we saw mCherry expressed from the *p^antidote long^-mCherry* reporter at a similar level to the mCherry expressed from the shorter *p^antidote^* (S7A–S7C Fig). The simplest interpretation of our data is that constructs under the *p^antidote^* promoter are expressed before and after spore formation. In addition, the pattern of reporter signal enrichment we observe suggests the transcripts and proteins produced in a spore can be retained within that spore. We did not directly explore a role of the 5’ UTR in ensuring spore-specific expression, but it is possible the UTR could affect translation or transcript retention in the spore.

For the *p^poison^-GFP* reporter, we observed expression in diploid cells induced to undergo meiosis. We also observed similar GFP reporter signal among the four spores (Fig 2E and 2F). These observations are similar to the localization patterns we observe with Wtf4^poison^-GFP (e.g., Fig 1C). This roughly equal distribution of *p^poison^-GFP* reporter signal in the four spores, even though only two inherited the *p^poison^-GFP* reporter, was starkly different from the two-spore enrichment observed with *p^antidote^*-*mCherry* reporter. Assuming equal spore to spore mobility between GFP and mCherry, this further supports that the majority of the the *p^poison^* transcripts are produced prior to spore individualization. As with the *p^antidote^*, it is possible that the 5’ UTR affects the translation or mobility of transcripts driven by *p^poison^* promoter, but we did not explore these possibilities.

Together, these experiments demonstrate differential gene expression patterns under the *p^antidote^* and *p^poison^* promoters. Our results also show that these different promoters largely contribute to the localization of Wtf4^poison^ protein within all spores and the enrichment of the Wtf4^antidote^ protein within the spores that inherit the *wtf4* locus.

### Master meiotic regulator, Mei4, controls Wtf4^poison^ expression

We next sought to explore transcriptional regulation of the *wtf4* transcripts. Previous work noted that the transcription of multiple *wtf* genes was controlled by the fork-head transcription factor Mei4, as *wtf* transcription is decreased if *mei4* is deleted and *wtf* gene expression is increased if Mei4 is overexpressed [61]. Mei4 controls the expression of hundreds of genes and is known as the master regulator of middle meiosis genes [50,51]. However, the prior study was performed before the discovery that *wtf* drivers can make two transcripts, so it was not clear which transcript Mei4 controls.

**Fig 3:**
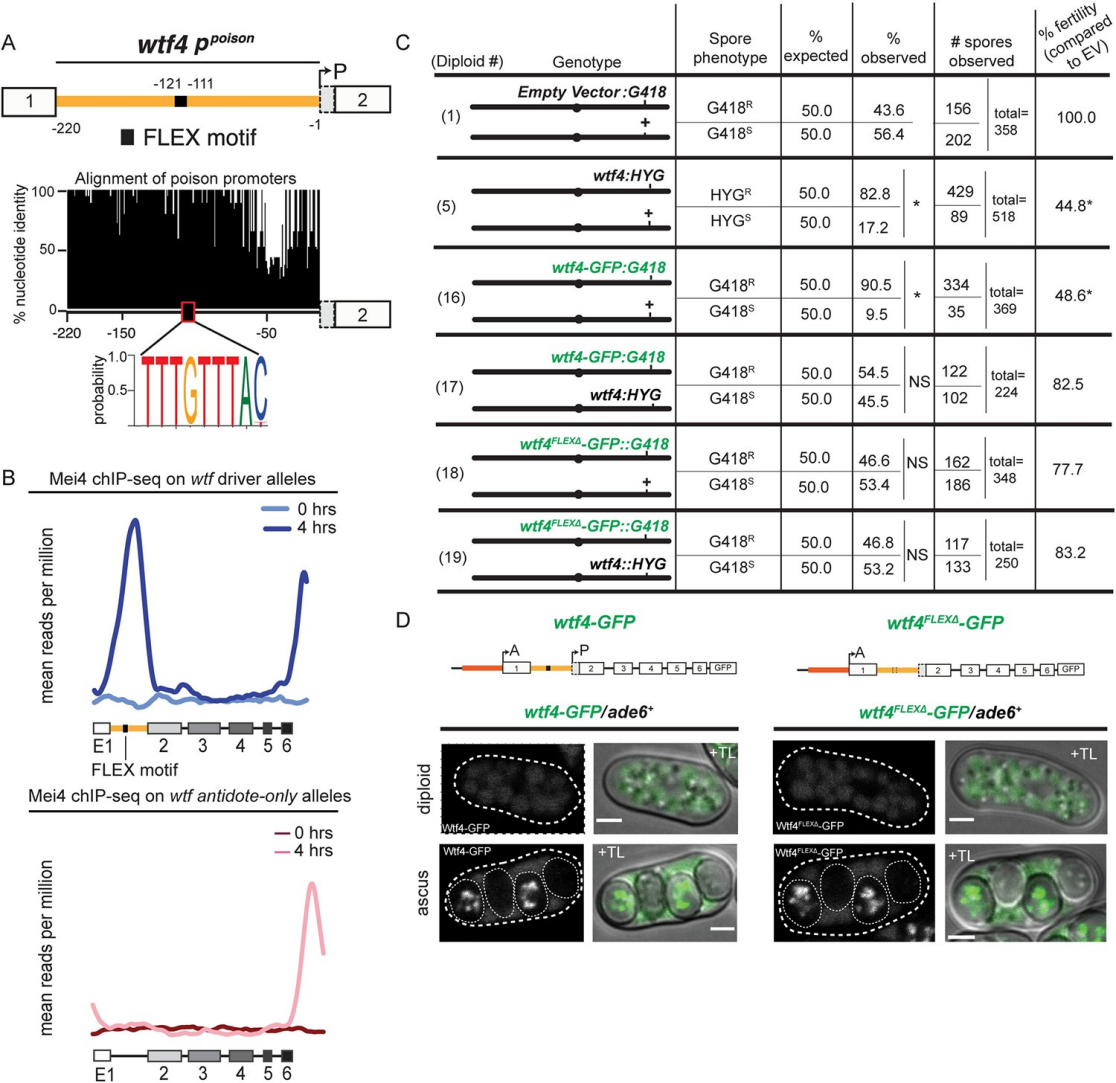
Master meiotic regulator, Mei4, controls Wtf4^poison^ transcription. (**A**) Depiction of the *wtf4*^poison^ promoter contained within intron 1. There is a FLEX motif (black box, TGTTTAC in opposite orientation) located 111 to 117 nucleotides upstream of the Wtf4^poison^ transcriptional start site. Percent consensus nucleotide identity of intron 1 sequences of 28 predicted poison-antidote *wtf* drivers [36] from three different strains of *S*. *pombe* (the reference genome, *S*. *kambucha*, and *FY29033*). The figure shows the percent consensus identity at each nucleotide position, excluding gaps. (**B**) Mei4 Chip-seq data (data from [50]) showing Mei4 binding on *wtf* genes at both 0 hours and 4 hours after meiotic induction. On the top panel, the reads are aligned to the predicted *wtf* meiotic drive genes (*wtf4*, *wtf13*) and on the bottom the reads are aligned to the *wtf* genes that are predicted to only encode antidote proteins (*wtf5*, *wtf9*, *wtf10*, *wtf16*, *wtf18*, *wtf20*, *wtf21*, *wtf25*) in the *S*. *pombe* genome version ASM294v2. For any reads that mapped to more than one location, only a single location, chosen at random, is reported. (**C**) Allele transmission and fertility (assayed via viable spore yield, VSY) of diploids with the depicted genotype. Alleles listed are at *ade6*. The genotype column shows a cartoon depiction of the relevant genotype. The progeny phenotypes are then shown on the right. For diploids heterozygous at one locus (e.g., Diploid 1), two values are shown (top and bottom) that represent the two possible haploid genotypes. Spores exhibiting both parental phenotypes were considered diploid or aneuploid and were excluded from this table but can be found in S1 Data. The expected values assume Mendelian allele transmission. (* = p < 0.05, NS = not significant; G-test for allele transmission, Wilcoxon test for VSY, in comparison to the empty vector control). We compared all diploids to diploid 1 as the control. The data for diploid 1 is also depicted in S2A and S9A Figs. Diploid 5 is also shown S2A Fig. (**D**) Images of heterozygous *wtf4-GFP/ade6+* (left) and *wtf4*^*FLEX*Δ^*-GFP/ade6+* (right) diploids and asci acquired after 1 day on sporulation media. Wtf4-GFP and Wtf4^FLEXΔ^ -GFP are shown in green. Scale bar represents 2 μM. TL = transmitted light. All images are shown at the same brightness and contrast for accurate comparison.

To understand which *wtf* transcript Mei4 controls, we first looked for the Mei4-binding motif in the *wtf4* promoters. Fork-head transcription factors bind sequences containing FLEX motifs and the complete Mei4 binding motif contains a nine-base pair (GTAAACAAA) core sequence [50,51,62,63]. We found this nine-base pair Mei4 binding motif in the *p^poison^* promoter 110 base pairs upstream of the *wtf4^poison^* translational start site (Fig 3A). Moreover, we found this sequence was conserved amongst known *wtf* drivers in *S*. *pombe* (Fig 3A) and in *wtf* drivers found in other *Schizosaccharomyces* species [64]. In contrast, the *p^antidote^* promoter does not contain a FLEX motif.

To test if Mei4 binds the *p^poison^* FLEX motif in meiotic cells, we analyzed data from a previous Chromatin-Immunoprecipitation sequencing (ChIP-seq) experiment of Mei4 during meiosis done in the lab isolate of *S*. *pombe* [50]. Due to the similarity of the *wtf* genes, many reads could not be uniquely assigned to *wtf4* or any single *wtf* gene and were thus randomly assigned to matching sites. The sequence of intron 1, however, is distinct between *wtf* drivers that contain the *p^poison^* promoter and antidote-only *wtf* genes that do not contain the *p^poison^* promoter, allowing us to distinguish Mei4 binding between intact *wtf* drivers and other *wtf* genes [36,38,39]. Prior to meiosis, we observed that Mei4 binding to *wtf* drivers (*wtf4*, *wtf13*) was low relative to the genome average (Fig 3B, 0 hours). After 4 hours in meiosis, when most cells are in prophase I, we saw a strong increase in reads in the *p^poison^* promoter (intron 1) of the *wtf* drivers (Fig 3B, 4 hours). We did not see an increase in Mei4 binding to antidote-only *wtf* genes (*wtf5*, *wtf9*, *wtf10*, *wtf16*, *wtf18*, *wtf20*, *wtf21*, and *wtf25*) during meiosis (Fig 3B). We also saw a Mei4 peak at the C-terminal region of both subsets of *wtf* genes. We predict this peak is due to the nearby LTRs, as we saw high Mei4 binding on LTRs independently of their association with *wtfs* (S8 Fig).

To further test the idea that *wtf4^poison^* expression is controlled by Mei4, we compared two tagged alleles: a fully functional allele (*wtf4-GFP*, [37]) and an allele lacking the FLEX motif (*wtf4*^*FLEX*Δ^*-GFP*; S1 Fig alleles 7 and 8). We integrated the alleles at *ade6* to test function. We did not anticipate that deletion of the Mei4-binding motif would affect the expression or function of the Wtf4^antidote^ protein as we can delete all of intron 1 from *wtf4* (as in *wtf4^antidote^-mCherry*), without affecting Wtf4^antidote^ function (S1 Fig allele 4, S2A Fig diploids 8 and 9). Consistent with our expectations, the *wtf4*^*FLEX*Δ^*-GFP* allele encodes a functional antidote as it suppresses drive of wild-type *wtf4* in a *wtf4*^*FLEX*Δ^*-GFP* /*wtf4+* heterozygote (Fig 3C, diploid 19). In addition, we saw strong GFP signal within two of the four spores in asci generated by *wtf4*^*FLEX*Δ^-GFP/*ade6+* diploids (Fig 3D), similar to tagged Wtf4^antidote^ alleles (e.g., S2C and S2D Fig). We tested the ability of the *wtf4*^*FLEX*Δ^*-GFP* allele to encode a functional Wtf4^poison^ by assaying if the allele could drive in a w*tf4*^*FLEX*Δ^-GFP/*ade6+* heterozygote. These diploids had Mendelian allele transmission (Fig 3C, diploid 18). As the w*tf4*^*FLEX*Δ^-GFP does encode a functional antidote, we conclude that the allele does not drive because the Mei4-binding motif is essential for production of the Wtf4^poison^. These data, combined with the previous genetic and biochemical data of others [50,61] support that Mei4 controls expression of the Wtf4^poison^ and likely the poison proteins produced by other *wtf* drivers.

### Distinct transcriptional regulation of Wtf4^poison^ and Wtf4^antidote^ promote efficient meiotic drive

To explore the functional relevance of the differential transcriptional of *wtf4^poison^* and *wtf4^antidote^*, we generated alleles that expressed the antidote protein from the *p^poison^* promoter and *vice versa*. We used the *p^poison^-wtf4^antidote^-GFP* allele (integrated at *ade6*), to test if the pattern of *p^antidote^* transcription was important for drive (S1 Fig allele 9). We compared the phenotype of this promoter swap allele to the functional *wtf4^antidote^-GFP* allele (integrated at *ade6*) with the endogenous promoter described above (S1 Fig allele 5). We found that the *p^poison^-wtf4^antidote^-GFP* was defective in meiotic drive. Specifically, the *p^poison^-wtf4^antidote^-GFP* allele was unable to suppress the drive of an intact *wtf4* driver in a *p^poison^-wtf4^antidote^-GFP/wtf4* heterozygote (S9A Fig diploid 22). The control allele (*wtf4^antidote^-GFP*) fully suppressed drive of intact *wtf4* (S9A Fig diploid 15). We also tested the ability of the *p^poison^-wtf4^antidote^-GFP* to suppress the spore inviability caused by an allele that expresses only Wtf4^poison^ (*mCherry-wtf4^poison^*; S1 Fig allele 10). We found that the *p^poison^-wtf4^antidote^-GFP* provided only minimal rescue of spore death and that the rescue was restricted to the spores that inherited the *p^poison^-wtf4^antidote^-GFP* (S9A Fig compare diploids 12 and 21).

Our results led us to question why Wtf4^poison^ expressed from the *p^poison^* promoter was able to poison all spores, but the Wtf4^antidote^ expressed from the *p^poison^* promoter was not able to rescue all spores. Such a rescue was expected as we previously observed that Wtf4^poison^ ectopically induced in mitotic cells is neutralized by expression of Wtf4^antidote^ from a matching promoter [52]. To explain this discrepancy, we hypothesized that the Wtf4^antidote^ may be more likely than the Wtf4^poison^ to be excluded from spores as they individualize within the ascal cytoplasm. This hypothesis was based on our images of Wtf4^antidote^ proteins with C-terminal tags that tended to show more signal in the ascal cytoplasm than within all newly developed spores. There also tended to be more signal in the ascal cytoplasm than within spores that did not inherit the locus encoding the Wtf4^antidote^ in mature asci (Figs 1E, S2C and S2D). If the Wtf4^antidote^ is excluded from spores as they individualize more than Wtf4^poison^, Wtf4^antidote^ would not be expected to protect spores well unless it was expressed in them.

**Fig 4:**
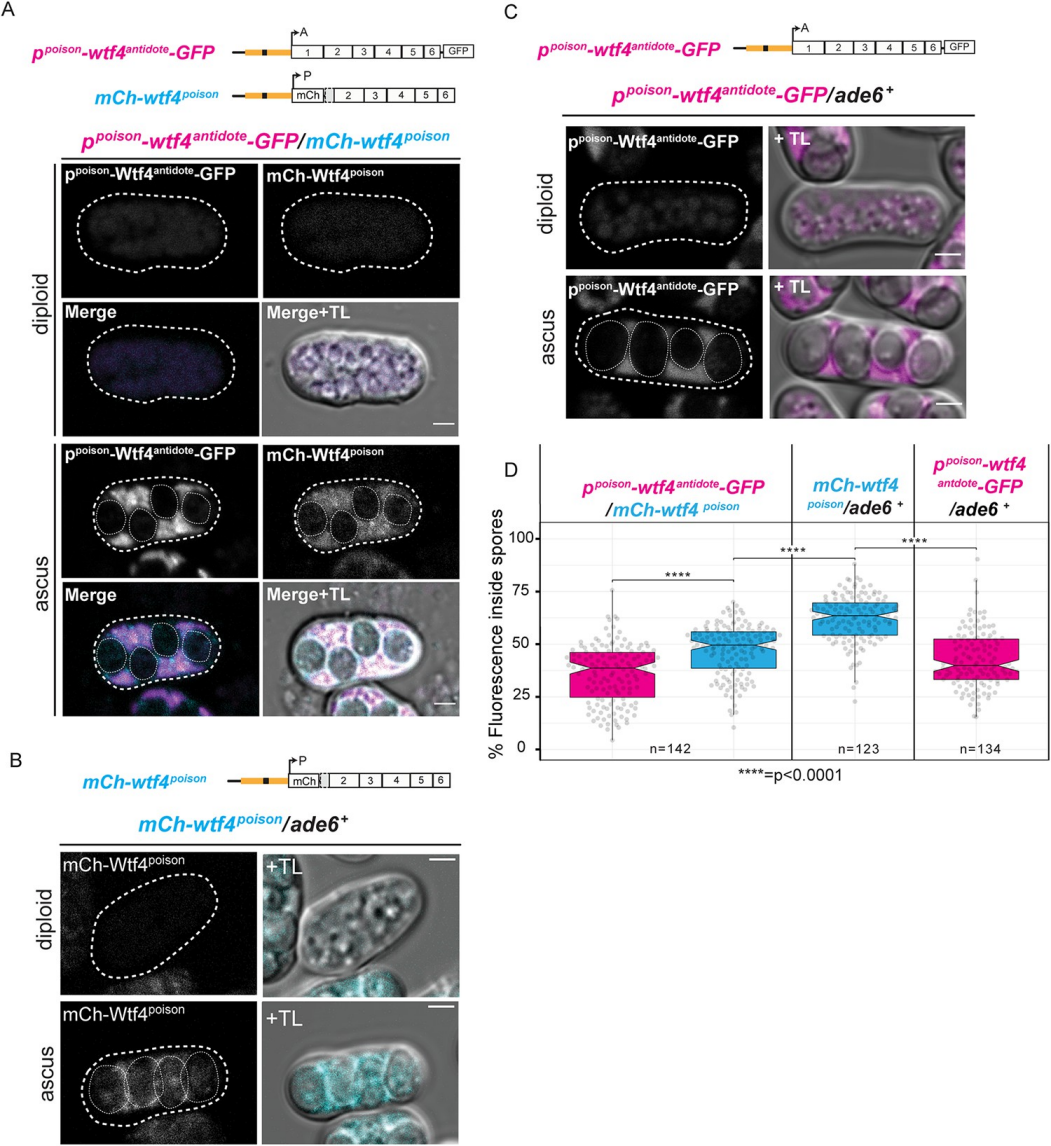
Wtf4^antidote^ is excluded from developing spores more than the Wtf4^poison^. (**A**) Image of a *p*^*poison*^-*wtf4*^*antidote*^*-GFP/ mCherry-wtf4*^*poison*^ diploid and ascus. (**B**) Image of a *mCherry-wtf4*^*poison*^*/ ade6+* diploid and ascus. (**C**) Image of *p*^*poison*^*-wtf4*^*antidote*^*-GFP*/ *ade6+* diploid and ascus. (**D**) Quantification of the percentage of the total fluorescence intensity found inside of spores of mature asci produced by *p*^*poison*^-*wtf4*^*antidote*^*-GFP/ mCherry-wtf4*^*poison*^ (left, n = 142), *mCherry-wtf4*^*poison*^*/ade6*^*+*^ diploids (center, n = 123) and *p*^*poison*^*-wtf4*^*antidote*^*-GFP/ade6*^*+*^ diploids (right, n = 134). p^poison^-Wtf4^antidote^-GFP fluorescence data is depicted in magenta while mCherry-Wtf4^poison^ is depicted in cyan. Error bars depict the 95% confidence interval. p^poison^-Wtf4^antidote^-GFP is shown in magenta and mCherry-Wtf4^poison^ is shown in cyan in merged images. TL = transmitted light. All scale bars represent 2 μm. All images were acquired after 1 day on sporulation media. Images were taken at the same settings and are shown at the same brightness and contrast for accurate comparison.

To formally test our hypothesis, we measured the fraction of the Wtf4^antidote^-GFP and mCherry-Wtf4^poison^ protein found within spores in asci generated by diploids carrying the *p^poison^-wtf4^antidote^-GFP* and/or the *mCherry-wtf4^poison^* allele (Fig 4A–4D). We found that a larger fraction of the mCherry-Wtf4^poison^ signal was in spores, relative to the fraction of total Wtf4^antidote^-GFP signal found in spores (Fig 4D, left panel). This suggests that the Wtf4^antidote^ protein is excluded from spores more than the Wtf^poison^ as spores individualize. In addition, we found that a smaller fraction of the mCherry-Wtf4^poison^ signal was in spores produced by diploids carrying the *p^poison^-wtf4^antidote^-GFP* allele, compared to diploids not expressing a *wtf4^antidote^* allele. This suggests the Wtf4^antidote^ can promote the exclusion of some Wtf4^poison^ from spores as they individualize.

To test if the *p^poison^* transcriptional timing was important for drive, we generated an allele with Wtf4^poison^-GFP expressed by the *p^antidote^* promoter (*p^antidote^-wtf4^poison^-GFP*; S1 Fig allele 11). However, we failed to transform this construct into cells not carrying a *wtf4^antidote^* allele, despite multiple attempts and using multiple transformation protocols (standard lithium acetate and electroporation; [65,66]). We were also unable to generate strains carrying *p^antidote^-wtf4^poison^-GFP* in the absence of *wtf4^antidote^* via mutation or genetic crosses (S9A Fig diploid 24). These results suggest that the *p^antidote^* promoter may cause some Wtf4^poison^ expression during vegetative growth, killing cells lacking *wtf4^antidote^.* Consistent with this, published long-read sequencing data shows some transcription of *wtf^antidote^* alleles occurs in cells prior to meiotic induction [59].

We assayed the ability of the *p^antidote^-wtf4^poison^-GFP* allele (at *ade6*) to support drive in cells heterozygous for *wtf4^antidote^-mCherry* (at *ura4*). We compared the *p^antidote^-wtf4^poison^-GFP* allele to a control *mCherry-wtf4^poison^* allele. We found that the *p^antidote^-wtf4^poison^-GFP* allele supported minimal drive of the *wtf4^antidote^-mCherry* allele, while the control *mCherry-wtf4^poison^* allele supported strong drive of *wtf4^antidote^-mCherry* (S9A Fig, diploids 23 and 24). Importantly most of the drive of the *wtf4^antidote^-mCherry* allele induced by *p^antidote^-wtf4^poison^-GFP* stems from the poison allele killing spores that inherit it (100% drive in spores that inherit *p^antidote^-wtf4^poison^-GFP)*, with minimal killing of spores that do not (58% drive in spores that do not inherit *p^antidote^-wtf4^poison^-GFP*). In the control cross, drive of the *wtf4^antidote^-mCherry* allele was 100% and 91% in the spores that did and did not inherit the *mCherry-wtf4^poison^* allele, respectively (S9A Fig, diploids 23 and 24).

**Fig 5:**
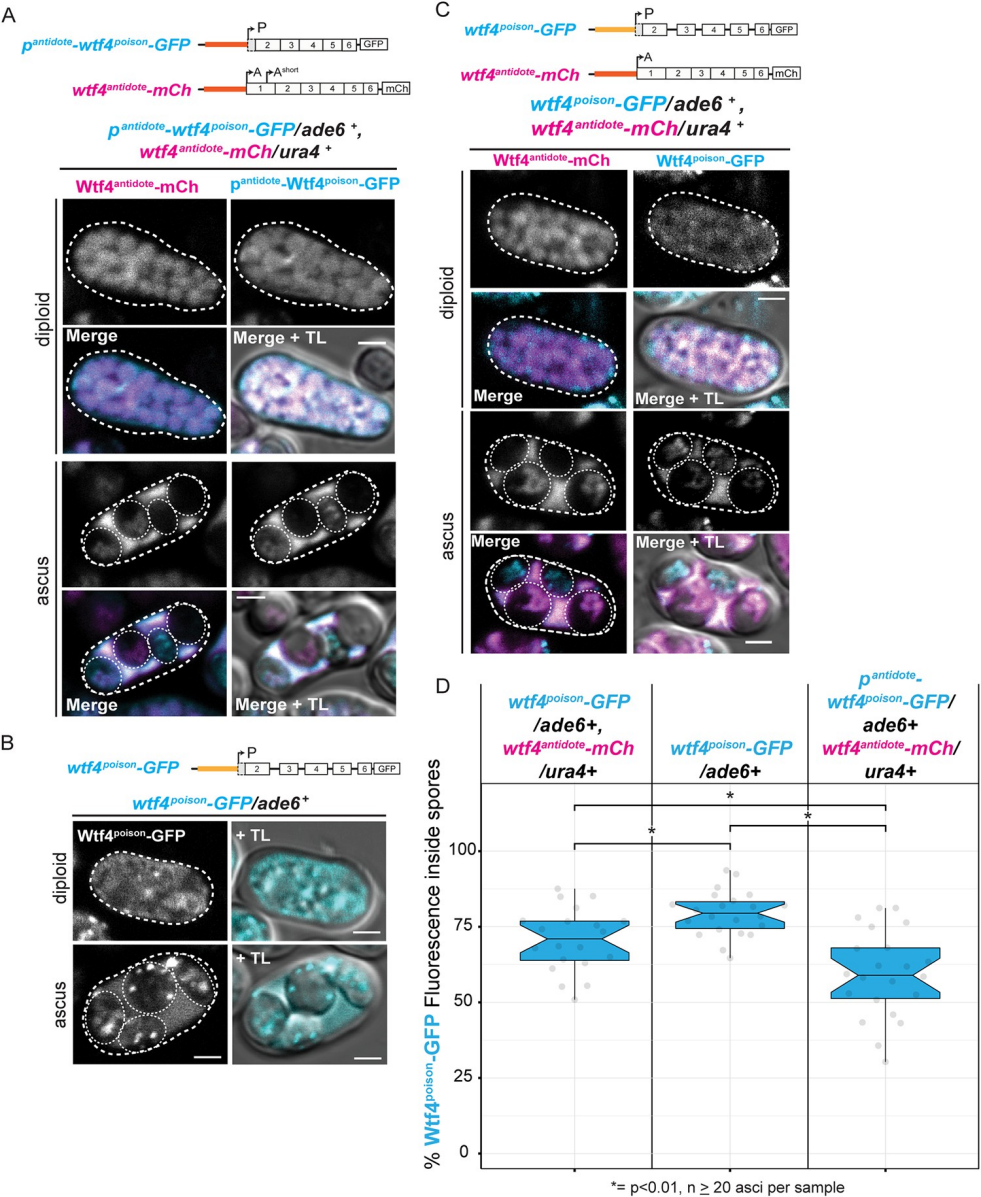
Wtf4^poison^ localization is altered when expressed from the *p*^*antidote*^ promoter. (**A**) Image of a *p*^*antidote*^-*wtf4*^*poison*^*-GFP/ ade6+*, *wtf4*^*antidote*^*-mCherry/ura4+* diploid and tetratype ascus. p^antidote^-Wtf4^poison^-GFP is shown in cyan and Wtf4^antidote^-mCherry is shown in magenta in merged images. Additional images (parental ditype and non-parental ditype) can be found in S9B Fig. (**B**) Image of a *wtf4*^*poison*^*-GFP/ ade6+* diploid and ascus. Images of this strain were first presented in [37]. (**C**) Images of a heterozygous *wtf4*^*antidote*^*-mCherry/ura4+*, *wtf4*^*poison*^*-GFP/ade6+* diploid and ascus. Wtf4^antidote^-mCherry is shown in magenta and Wtf4^poison^-GFP is shown in cyan in merged images. Images of this cross are also depicted in Fig 1E. (**D**) Quantification of the percent of total ascal Wtf4^poison^-GFP fluorescence located outside spores in asci generated by the following diploids: 1. *wtf4*^*poison*^*-GFP/ade6+*, *wtf4*^*antidote*^*-mCh/ura4+*, 2. *wtf4*^*poison*^*-GFP/ade6+*, and 3. *p*^*antidote*^*-wtf4*^*poison*^*-GFP/ade6+*, *wtf4*^*antidote*^*-mCh/ura4+* (* = p < 0.01, t-test, n> 20 asci per sample). TL = transmitted light. All scale bars represent 2 μm. All images were acquired after 2 days on sporulation media. Images were taken using the same settings. Not all images are shown at the same brightness and contrast to avoid over saturation of pixels in the brighter images.

Our genetic results indicate that the Wtf4^poison^ expressed from the *p^antidote^* promoter is defective at killing spores that do not inherit the allele encoding the Wtf4^poison^. This is surprising given the high sensitivity of mitotic *S*. *pombe* cells to even low levels of Wtf4^poison^ we observed in previous work [52] and in this study (e.g., S9 Fig Diploid 24). We hypothesized that the Wtf4^antidote^ is better at preventing Wtf4^poison^ from entering spores when the proteins were co-expressed from the p^antidote^ promoter than when the Wtf4^poison^ is expressed from its endogenous promoter. To test this possibility, we compared the amount of Wtf4^poison^ signal in spores produced by diploids heterozygous for both *p^antidote^-wtf4^poison^-GFP* allele (at *ade6*) and *wtf4^antidote^-mCherry* (at *ura4*) to similar control diploids in which *wtf4^poison^-GFP* was expressed from its endogenous promoter (Fig 5A and 5B). Consistent with our hypothesis, we found that less of the total Wtf4^poison^-GFP signal was inside of spores when the protein was expressed from the *p^antidote^* promoter, as compared to when the protein was expressed from the endogenous *p^poison^* promoter (59 vs 71%). We also noticed that less Wtf4^poison^-GFP (from the endogenous promoter) was included spores in the presence of the Wtf4^antidote^ protein (71 vs 79%), further supporting that the Wtf4^antidote^ plays a role in excluding Wtf4^poison^ from spores (Fig 5B–5D).

## Discussion

### Dual promoters with distinct regulation are key to *wtf* meiotic drive

In *wtf4* drive, all spores are exposed to the Wtf4^poison^, while only those that inherit the *wtf4* allele generally receive a sufficient dose of the Wtf4^antidote^ to survive. Previously, we observed Wtf4^poison^ in meiotic cells prior to spore formation, but observed Wtf4^antidote^ enriched only in spores that inherited *wtf4* [37]. These observations explained how all spores were poisoned and how only those that inherited the *wtf4* locus were rescued by the antidote, but it raised a new puzzle of how meiotic cells survived exposure to the Wtf4^poison^ in the apparent absence of the Wtf4^antidote^. This uncertainty was amplified by subsequent work that showed Wtf4^poison^ efficiently kills cells, not just spores, that do not express antidote [52]. In this work, our new data offer a solution to the puzzle in that we found there is also Wtf4^antidote^ expression in cells undergoing meiosis. This result is consistent with previous long-read RNA sequencing data that show a low level of transcription of *wtf^antidote^* alleles in early meiosis [59].

Our work also reveals that different transcriptional regulation of the poison and antidote promoters is largely responsible for the distribution of the Wtf4^poison^ to all spores and the enrichment of the Wtf4^antidote^ in the spores that inherit the locus, as transcriptional reporters grossly recapitulate localization patterns of tagged proteins (Fig 2). However, different features of the proteins also contribute to their localization patterns. Specifically, we found that the propensity for the two Wtf proteins to be included in developing spores was different. The Wtf4^antidote^ present in the cytoplasm at the time of spore encapsulation is excluded from the spores more than Wtf4^poison^ present at the same time (Fig 4D). In addition, our results suggest that Wtf4^antidote^ protein can promote exclusion of the Wtf4^poison^ from spores as they individualize, as a smaller fraction of the Wtf4^poison^ enters spores in the presence of the Wtf4^antidote^ (Figs 4D and 5D). This change in localization of the Wtf4^poison^ in the presence of the Wtf4^antidote^ is consistent with our previous observations that the Wtf4 proteins co-assemble and are trafficked to the vacuole [52].

**Fig 6:**
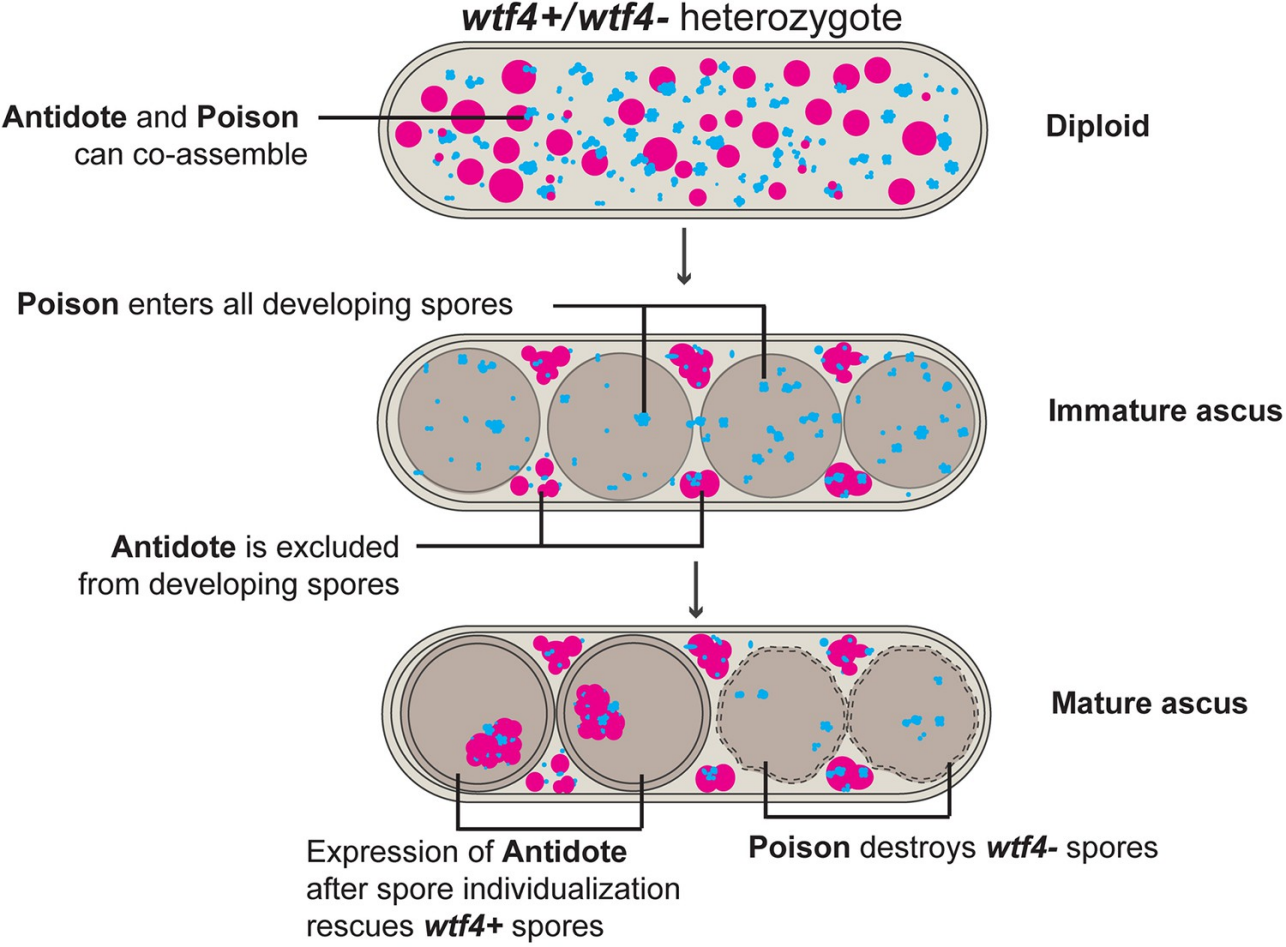
Model of *wtf4* drive in *wtf4*+/*wtf4*- heterozygote. The Wtf4 proteins are both present prior to spore formation (top). As spores form (middle), more Wtf4^antidote^ is excluded from spores relative to Wtf4^poison^. Additional Wtf4^antidote^ is produced in *wtf4*+ spores (bottom), which rescues them from the Wtf4^poison^. The *wtf4*- spores are destroyed by the Wtf4^poison^.

Our data suggest the following model (Fig 6): the Wtf4^poison^ is mostly expressed prior to spore formation and then subsequently packaged in spores (e.g., Fig 1E). This likely ensures that each spore gets a lethal dose from a heterozygous zygote. Conversely, Wtf4^antidote^ is present prior to spore formation, perhaps to prevent the cells undergoing meiosis from succumbing to the poison (e.g., Fig 1E). The antidote that is present at the time of spore formation likely offers little protection to spores that do not inherit the driving locus because it does not prevent all Wtf4^poison^ from entering spores (Fig 5D) and the Wtf4^antidote^ is excluded from developing spores (Fig 4). We speculate that the exclusion of Wtf4^antidote^ from spores is due to its association with vacuoles, which are also excluded from developing spores [52,67]. Because of these factors, spores that inherit *wtf4* must produce their own private supply of Wtf4^antidote^, while those that do not succumb to the poison.

### Difficulties in universal suppression of *wtf* drivers

This work also adds to our understanding of the evolutionary success of the *wtf* drivers within *Schizosaccharomyces* species [64]. In *S*. *pombe*, all sequenced isolates contain between 25 and 38 *wtf* genes, including 4–14 genes that are predicted or shown to be intact meiotic drivers [35–38]. Each heterozygous driver can cause the destruction of half the spores and antidote proteins generally do not protect against poisons that have distinct sequences [35,38]. Because of these features, diploid zygotes produced by crossing two diverged isolates produce very few viable spores [35,38,43–45,68,69]. The incentive to evolve a suppression mechanism to suppress a *wtf* driver, therefore seems quite high [70].

There are likely many roads to suppression of *wtf* drivers. We speculate, however, that none of them have been terribly successful given the *wtf* gene family has been causing meiotic drive for over 100 million years [64]. The known genic suppressors of *wtf* drivers are all Wtf^antidote^ proteins. Importantly, Wtf^antidote^ proteins seem highly specific in that they neutralize only Wtf^poison^ proteins with very similar sequences [35,38,39,52]. Because of this, Wtf^antidote^ proteins are not expected to act as general suppressors of *wtf* drivers, as the genome would require a unique suppressor for each driver. A non-Wtf universal suppressor that interfaces with Wtf^poison^ proteins might also be difficult to envision given the vast diversity of Wtf proteins, which can share as little as 30% amino acid identity within a species [35,36].

Transcriptional repression seems like a more feasible route to universal suppression of *wtf* drivers given the conservation of their promoters ([36,38,60]; Fig 2A and 2D). Previous work has identified some factors that help regulate the expression of *wtf* genes, however, these studies did not distinguish between *wtf^poison^* and *wtf^antidote^* transcripts [46,48]. In mitotic cells, a mutation in the histone deacetylase *clr6* or inhibition of histone deacetylation with the drug Trichostatin A leads to increased *wtf* gene transcription [46–48,71]. Also in mitotic cells, *wtf* mRNAs can be eliminated from cells via double-stranded-directed RNA decay [72]. It is possible that these mitotic mechanisms may affect *wtf* gene expression during meiosis as well, but this has not been determined. In meiotic cells, Cuf2 decreases the expression of *wtf* genes late in meiosis [51,73]. However, this transcriptional suppression appears to have a minimal effect on drive. For example, Cuf2 decreases expression of *wtf13*, but even in this suppressed state, *wtf13* drives into >90% of the gametes produced by a *wtf13+/wtf13*Δ heterozygote [39].

It is important to note that not all *wtf* genes are equally expressed, and differential expression of the *wtf^poison^* transcripts is correlated to drive strength in *S*. *octosporus* [37,38,59,64]. This differential expression could reflect an inefficient transcriptional repression system, but that has not been investigated. Still, effective transcriptional repression of all *wtf* drivers could be challenging for several reasons. The first challenge is the large number of *wtf* genes and the fact that they are not all found at the same locus. This may make it hard to simply turn them all off *en masse* by packaging one genomic region in heterochromatin. Even if the genes were found in one location, the genomic region housing the *wtf* genes would likely be under strong selection to resist such heterochromatization. Specifically, haplotypes that could avoid heterochromatization and thus better maintain drive would be selected by drive. This is because regions linked to meiotic drivers also get to enjoy the benefits of drive and thus profit from enhancing drive, rather than suppressing it [70]. This selective pressure resisting heterochromatization would also apply to the distributed *wtf* drivers as well. This situation is profoundly different than loci housing transposable elements as those selfish elements generally offer no evolutionary advantages to flanking sequences [2].

Another challenge in establishing suppression of *wtf* drivers is that partial or imperfect suppression of *wtf* genes may do more harm than good. For example, if silencing is incomplete in a diploid homozygous for a given driver, only one allele could be expressed, and drive would occur in a cell where no drive would occur in the absence of silencing. In addition, in some instances *wtf* drivers are suppressed by other *wtf* genes [35,39]. This is an additional situation in which incomplete silencing of the wrong *wtf* gene could lead to more, rather than less, drive.

An ideal suppression approach would be to suppress a key transcription factor driving expression from *p^poison^* promoters. This approach would allow the cell to stop drive, but it also comes with minimal risk as partial suppression would still be selectively advantageous by decreasing the likelihood of drive. In this scenario, an imperfect system could adapt and improve over time. In this work, we identified Mei4 as the key transcription factor governing the expression of *p^poison^* (Fig 3). Suppression of Mei4, however, is not an ideal option for suppressing drive because it is an essential regulator of meiosis. Mei4 controls the expression of over 100 genes and cells lacking Mei4 fail to complete meiosis [50,51,62,63,74–77].

### Final speculation

It is hard not to admire how beautifully adapted the *wtf* parasites are at promoting themselves despite the costs. Equally remarkable is how well insulated *wtf* parasites appear to be against their host genomes acting to stop their expression. That is not to say, however, that *Schizosaccharomycetes* have not found other ways to mitigate the costs of *wtf* drivers. Some *S*. *pombe* natural isolates preferentially inbreed and some isolates generate disomic (aneuploid or diploid) spores at high frequencies (up to 46% of spores; [41,78]). Although it is hard to say if the *wtf* genes promoted the evolution of these traits, it is possible, as both traits effectively suppress fitness costs of *wtf* drivers [41,78]. If these traits were selected for based on their ability to suppress drive, suppression likely came at a steep price to fitness, given that inbreeding and meiotic disruption are generally not good for fitness. Perhaps this was the price that had to be paid, as cheaper options, like universal transcriptional silencing, were out of reach.

## Materials and methods

### Generation of yeast strains

All yeast strains, plasmids, and oligos are described in S1–S3 Tables. We confirmed the plasmids generated in this section via Sanger sequencing. We completed all transformations described in this section using standard lithium acetate protocol [65], first selecting for drug resistance and then screening for the relevant auxotrophy.

#### Generation of an allele of *wtf4* with endogenous promoters and *mCherry-wtf4^poison^*

We ordered a gBlock from Integrated DNA technologies (IDT, Coralville IA) containing the *p*^antidote^ promoter (-600 bp), exon 1, intron 1, and sequence encoding an mCherry tag and a 5X glycine linker upstream of the *wtf4^poison^* start codon [79]. The gBlock also included 177 bp of exon 2. We amplified this gBlock using oligos 620 and 718. We also amplified the rest of the *wtf4* sequence from pSZB189 using oligos 679 and 687 [37]. We then used overlap PCR to stitch the PCR fragments together using oligos 620 and 687. Next, we digested the resulting PCR product with SacI and ligated it into the SacI site of pSZB188 [37], an *ade6* integrating vector with a *kanMX4* cassette, to create pSZB250. We cut pSZB250 with KpnI and transformed the linearized vector into SZY643 to generate SZY1037.

#### Generation of a *wtf4* allele that encodes only mCherry-Wtf4^poison^ under the control of the endogenous promoter

This allele has the full-length *wtf4* gene (including *p^antidote^* promoter) but the two start codons in exon 1 (for the Wtf4^antidote^) are mutated to TAG. We first cloned pSZB259 which contains an untagged *wtf4* allele in which both start codons are mutated, similar to pSZB257 [37]. We amplified the *p^antidote^* promoter and the mutated version of exon 1 from pSZB259 using oligos 620 and 861. We amplified the *mCherry-wtf4^poison^* sequence from pSZB250 (described above) using oligos 862 and 687. We then used overlap PCR to stitch the two PCR products together using oligos 620 and 687. We digested the resulting PCR product with SacI and ligated the cassette into the SacI site of pSZB188 [37] to make pSZB355. We also digested pSZB355 with SacI to isolate the *wtf4 allele* and cloned it into SacI-digested pSZB386, a hygromycin B-resistant *ade6* integrating vector [37] to generate pSZB824. We digested pSZB824 with KpnI to linearize the construct and transformed into SZY2572 to generate SZY4570.

#### Generation of a *wtf4* allele that encodes only Wtf4^antidote^-GFP under the control of the endogenous promoter

We used pSZB203 as a template to amplify the 5’ end of *wtf4-GFP* with oligos 620 and 736 and the rest of the gene with oligos 735 and 634 [37]. The two PCR products were then stitched together using overlap PCR using oligos 620 and 634. The 735 and 736 oligos mutated the start codon for the *wtf4^poison^* to TAC. The resulting PCR product was then digested with SacI and ligated into the SacI site of pSZB188 to generate pSZB260. We cut pSZB260 with KpnI and integrated it into the *ade6* locus of SZY643 to create SZY1056.

#### Generation of a *wtf4* allele that encodes only mCherry-Wtf4^antidote^ under the control of the endogenous promoter

We used site-directed mutagenesis to mutate the translational start site from ATG to TAC of *wtf4^poison^* within pSZB248 [37] to generate pSZB367. We linearized pSZB367 with KpnI and integrated it into SZY643 to make SZY3586.

#### Generation of a *wtf4* allele with the first antidote start site mutated to make the *wtf4^short^* allele

This allele has the full length *wtf4* gene, with the first antidote start site (ATG) mutated to TAG. Using pSZB189 as a template, we amplified the 5’ end of *wtf4* with the mutated start site using oligos 620 and 702. We then amplified the rest of the *wtf4* gene using oligos 701 and 686 with pSZB189 as the template. The two PCR products were then stitched together using overlap PCR using oligos 620 and 686 and ligated into the SacI site of pSZB188 to generate pSZB244. We cut pSZB244 with KpnI and integrated it into the *ade6* locus of SZY643 to create SZY1026.

#### Generation of a p^antidote^-mCherry transcriptional reporter

We first amplified the *wtf4^antidote^* promoter linked to mCherry using pSZB248 [37] as a template with oligos 688 and 1447. Next, we amplified the *ADH1* transcriptional terminator from pKT127 [80] using oligos 1448 and 634. We then used overlap PCR to unite these fragments and generate *p^antidote^-mCherry*. We then digested the complete *p^antidote^-mCherry* cassette with SacI and ligated it into the SacI site of pSZB386 [39] to create pSZB766. We cut pSZB766 with KpnI and integrated it into the *ade6* locus of SZY2080 to create SZY2137. We also digested the cassette form pSZB766 and cloned it into SacI-digested pSZB331 [35] to create pSZB744. We cut pSZB744 with KpnI and integrated it into the *ura4* locus of SZY2080 to create SZY4534.

#### Generation of a p^antidote long^-mCherry transcriptional reporter

We amplified the majority of the *p^antidote long^-mCherry* construct (with an *ADH1* transcriptional terminator) using oligos 3024 and 634 and pSZB891 [52] as a template. We then added the rest of the upstream to the *p^antidote long^-mCherry* construct by using it as a template for PCR with oligos 3025 and 634. This generated the full *p^antidote long^-mCherry* construct. We digested this cassette with SacI and ligated it into the SacI site of pSZB322 [39] to create pSZB1361. We cut pSZB1361 with KpnI and integrated into the *lys4* locus of SZY2080 [52] to generate SZY4442.

#### Generation of a strain with *wtf4::hphMX6* at the *ade6* locus

We digested pAG32 (*Goldstein and McCuster*, *1999*) with NotI to isolate the *hphMX6* cassette. We transformed the *hphMX6* cassette into SZY887 [37] selecting for HYG resistance and lack of G418 resistance. This created SZY969.

#### Generation of a p^poison^-GFP transcriptional reporter

We used pSZB203 as a template to amplify the *p^poison^* promoter using oligos 1174 and 1549, and to amplify GFP (with an *ADH1* transcriptional terminator) using oligos 1548 and 634 [37]. We used overlap PCR to join these two pieces using oligos 1174 and 634. We then digested the complete *p^poison^-GFP* construct with SacI and cloned it into SacI-digested pSZB386 to generate pSZB821. We linearized pSZB821 with KpnI and integrated it into SZY643 to make SZY2279.

#### Generation of an allele of *wtf4^antidote^-GFP* in which the p^antidote^ promoter is replaced with the p^poison^ promoter

We amplified the poison promoter and exon 1 from pSZB553 (see above) using oligos 1174 and 604. We amplified the rest of the *wtf4* coding sequence from pSZB700 [52] using oligos 605 and 997. We amplified GFP (with an *ADH1* transcriptional terminator) from pSZB203 [37] using oligos 998 and 634. We used overlap PCR to join the three pieces using oligos 1174 and 634.We digested the complete *p^poison^-wtf4^antidote^-GFP* construct with SacI and cloned it into SacI-digested pSZB386 to generate pSZB727. We digested pSZB727 with KpnI to linearize the construct and transformed into SZY44 to generate SZY2406.

#### Generation of the *p^antidote^-wtf4^poison^-GFP* allele

We amplified *p^antidote^* from pSZB203 using oligos 688 and 1383 [37]. We amplified the *wtf4^poison^* coding sequence from pSZB392 [52] using oligos 1384 and 997. We amplified GFP (with an *ADH1* transcriptional terminator) from pSZB203 [37] using oligos 998 and 634. We used overlap PCR to join the three pieces using oligos 688 and 634. We digested the complete *p^antidote^-wtf4^poison^-GFP* construct with SacI and cloned it into SacI-digested pSZB386 to generate pSZB758. We digested pSZB758 with KpnI to linearize the construct and transformed into SZY2572 to generate SZY4525. The *p^antidote^-wtf4^poison^-GFP* allele lacks exon 1 and thus does not encode Wtf4^antidote^.

#### Generation the *wtf4^FLEXΔ^-GFP* allele

We used site-directed mutagenesis to delete the FLEX motif (TTTGTTTAC, [62,63]) within intron 1 of the *wtf4-GFP* allele in pSZB203 [37]. This generated pSZB848. We digested pSZB848 with KpnI to linearize the construct and transformed into SZY643 to generate SZY1479.

#### Generation of an *ade6+::his5Δ* allele

This was completed in the same manner as in [37]. Briefly, we amplified from genomic DNA a region upstream of *his5* using oligos 795 and 796, a region downstream of *his5* using oligos 797 and 798, and *ade6+* using oligos 799 and 800. We stitched these pieces together using overlap PCR and oligos 795 and 798. We then transformed the cassette into SZY631 [39], selecting for *ade6+* and then screening for *his5-*. This generated SZY1285 used in this work.

#### Generation of a *ura4+*, *wtf4^poison^-GFP* strain

We amplified a *ura4+* cassette using oligos 34 and 37 and SZY44 [37] as a template. We then transformed it into SZY1049 [37], selecting for *ura4+*, to generate SZY5047.

#### Generation of a *pat-1as(L95G)*, *wtf4^poison^-GFP* strain

We crossed SZY44 with J1687 [63] and selected for progeny that were resistant to hygromycin and G418, but were sensitive to nourseothricin, to obtain SZY5581. We then transformed KpnI-digested pSZB257 [37] into SZY5581, which resulted in SZY5638, a strain with *pat1.L95G and wtf4^poison^-GFP* alleles.

#### Generation of a *pat1.L95G, wtf4^antidote^-mCherry* strain

We transformed KpnI-digested pSZB891 [37] into SZY5581 to obtain SZY5685, a strain with the *pat1.L95G and wtf4^antidote^-mCherry* alleles.

#### Generation of a diploid with *pat1.L95G*, *wtf4^antidote^-mCherry*, *wtf4^poison^-GFP*

We generated *h-/h-* diploids (SZY5742 and SZY5743) by protoplast fusion of SZY5685 and SZY5638 as previously described [81], with the following changes: We grew 50 mL YEL cultures of each strain to mid-logarithmic phase (~5x10^6^ cells/ml) before washing in 10 mL 0.65 M KCl. We then prepared protoplasts by incubating cell pellets in 0.65 M KCl containing 0.1 g/mL Lallzyme MMX (Scott Labs) for 14 minutes. We then plated the protoplasts to YNP -adenine, -histidine, -leucine, -uracil and incubated at 32°C for 3 days. We then replica plated the colonies and imaged the cells to confirm genotypes and ploidy.

### Allele transmission and viable spore yield

All allele transmission and viable spore yield (VSY) assays were completed as previously described [37,53]. Briefly, we generated stable diploids by mixing each haploid parent in a microcentrifuge tube and plating them on SPA (1% glucose, 7.3 mM KH_2_PO_4_, vitamins, agar) for ~15 h at room temperature to allow the cells to mate. We scraped the mated cells off of SPA and spread on a medium to select for heterozygous diploids (minimal yeast nitrogen base plates). We grew diploid colonies overnight in 5 mL of rich YEL broth (0.5% yeast extract, 3% glucose, 250 mg/L of adenine, lysine, histidine, and uracil). We then plated ~100 mL onto SPA to induce sporulation, as well as diluted samples onto YEA (same as YEL, but with agar). We confirmed the colonies that grew on the YEA plate were truly heterozygous diploid cells by replicating to diagnostic media and counted the colonies for VSY. After 3 days, we scraped the cells from the SPA plates, treated it with glusulase (Sigma (G7017-10ML) and ethanol to isolate spores, and plated dilutions of the spores on YEA [53]. We then counted and phenotyped the spore colonies using standard approaches. At least 2 diploids were assayed per cross and at least 150 spores were genotyped for allele transmission assays. The raw data can be found in the S1 Data file.

### Alignment of promoters

For the alignment of antidote promoters (Fig 2A), we aligned the 800 base-pairs upstream of 41 predicted antidote-only alleles [36] from three different strains of *S*. *pombe* (the reference genome, *S*. *kambucha*, and *FY29033*, *Lock et al*., *2018*). For the alignment of poison promoters (Fig 2D), we aligned the intron 1 sequences (flanked by sequences 100bp upstream and downstream intron 1) of 28 predicted poison-antidote *wtf* drivers [36] from three different strains of *S*. *pombe* (the reference genome, *S*. *kambucha*, and *FY29033*). We utilized Geneious (version 11.0.14.1, https://www.geneious.com) using the Geneious aligner with the “global alignment without free end gaps” setting, taking the percent identity at each nucleotide position.

### Protein analysis with western blots

To isolate proteins for *pat1+* western blots (S4 and S5 Figs), we grew diploids ~20 hours to saturation in 10 ml YEL at 32°C. We then diluted cells 1:200 in 100 mL PM media and again grew at 32°C overnight (20–24 hours). We then washed the cells once in PM-N and then diluted to an O.D. 600 of 1.0 (~1x10^7^ cells/ml) in 500 ml PM-N incubated at 25°C. We prepared whole cell extracts at the time-points indicated in figure legends from 100 mL aliquots as described in [82] with the following addition. To block protein degradation, we added 1 mM PMSF to each time-point prior to spinning down cells. The cell lysis buffer (50 mM Tris-HCl pH 7.5, 150 mM NaCl, 1 mM EDTA, 0.5% NP-40, 10% Glycerol) we used was supplemented with 1 mM PMSF and 1 complete Mini, EDTA-free protease inhibitor cocktail tablet per 5 mL. We disrupted the cells using a Mini Bead beater (Biospec Products) and we imaged cells to ensure spores were broken.

To prepare positive controls expressing constitutive GFP or mCherry, we grew strains SZY2636 (GFP) and SZY2638 (mCherry) [78] to saturation in YEL, then pelleted a 1 mL volume, washed the cells in ddH_2_0, resuspended in 1 mL ddH_2_0 and plated 200 μl onto each of three SPA+S plates. We then incubated the plates at 25°C for three days and then scraped cells into 1 mL ice-cold PBS and prepped whole cell extracts as described above. We imaged the cells to ensure sporulation using a Zeiss Observer.Z1 wide-field microscope with a 63x 1.4 Oil DICII objective and collected the emission onto a Hamamatsu ORCA Flash 4.0 using μManager software. We used BP 440–470 nm to excite GFP and collected BP 525–550 emission using a FT 495 dichroic, and mCherry with BP 530–585 nm excitation and LP 615 emission, using an FT 600 dichroic filter.

To run western blots, we diluted samples 1:1 in LDS sample buffer (Life Technologies) and heated them for 10 min at 75°C before loading. We then ran the proteins on NuPAGE 4–12% Bis-Tris protein gels (Life Technologies) in 1 x MOPS buffer (Life Technologies) for 50 minutes at 200V and then transferred to PVDF membranes (Bio-RAD #1704156) using the Trans-Blot Turbo system. We probed the membranes with either a rabbit monoclonal, α-GFP antibody (Cell Signaling Technology #2956) at 1:1000 and/or a mouse monoclonal, α-mCherry antibody (Millipore, MAB131873) at 1:1000, overnight at 4°C with agitation in Odyssey blocking buffer (TBS, from LI-COR biosciences). Secondary, α-rabbit (800 cW) and/or α-mouse antibodies (680 cW) were used for fluorescent visualization of the proteins. We imaged the blots on the Odyssey-CLx (LI-COR biosciences). To show protein loading we either stained membranes with Ponceau S (Cell Signaling Technology #59803) or ran a second gel with the same volume of sample and stained with Imperial protein stain (Thermo Scientific #24615).

### Synchronization of *pat1*.*L95G* cultures

To generate synchronous meiotic cultures (S6 Fig), we grew *h-/h- pat1*.*L95G/pat1*.*L95G* analog-sensitive diploid strains (SZY5742 and SZY5743) for 20 hours at 30°C in 10 mL YEL media. We then diluted the cells 1:250 in 50 mL EMM supplemented with 75 μg/ml lysine and grew them for 20–24 hours at 30°C. We then washed the cells twice in EMM-N and diluted to an O.D. 600 of 1.0 in EMM-N supplemented with 10 μg/ml lysine. After 12 hours at 30°C, we added 50 μg/ml lysine, 500 μg/ml NH4Cl and 25 μM 3MB-PP1 (Cayman Chemicals) to each culture, and then sampled cells for DAPI staining, imaging and Westerns at the timepoints described in the figures.

### DAPI staining

We pelleted a 1 ml volume of each culture at the indicated timepoints and resuspended the cells in 1 ml 70% ethanol. We then incubated the cells at room temperature for one hour and then washed once in 1 ml PBS. We then immediately visualized cells using a Zeiss Observer.Z1 wide-field microscope with a 63x 1.4 Oil DICII objective and collected the emission onto a Hamamatsu ORCA Flash 4.0 using μManager software. We excited DAPI at 365 nm and fluorescence was collected through a 445/50 nm bandpass filter.

### Fluorescence microscopy

The raw data for all image quantification is presented in the S2 Data. We generated diploids as previously described [37] and placed them on sporulation agar (SPA, 1% glucose, 7.3 mM KH_2_PO_4_, vitamins, agar) for 1–3 days. We then scraped the cells off the plates and onto slides for imaging. For the *pat1*.*L95G* time course experiment shown in S6 Fig, we fixed the cells in 4% paraformaldehyde for one hour at room temperature, washed three times in 1 mL PBS and stored the cells at 4°C prior to imaging. For all microscopy, except for the experiments listed below, we used an LSM-780 (Zeiss) microscope, with a 40x C-Apochromat water-immersion objective (NA 1.2), in photon-counting channel mode with 488 and 561 nm excitation. We collected GFP fluorescence through a 481–552 bandpass filter and mCherry through a 572 long-pass filter. We also used photon-counting lambda mode, with 488 and 561 nm excitation, collecting fluorescence emission over the entire visible range. We then used these images to linearly unmix the fluorescence spectra using an in-house custom written plugin for ImageJ (https://imagej.nih.gov/ij/) to verify that there was no auto-fluorescence in the cells. Brightness and contrast are not the same for all images. We utilized two independent progenitor diploids and assayed at least 25 asci for each genotype represented. We called an ascus “mature” if all four spores showed distinct, dark outlines via transmitted light, suggesting spore membranes had been formed. For images where fluorescence intensity inside of spores is compared to fluorescence intensity outside of spores, great care was taken in acquiring the data so that only asci where all spores were sharply in focus were used for comparison.

For the *wtf4^antidote^-mCherryh/ura4+* (S2C Fig) we imaged using a Zeiss Observer.Z1 wide-field microscope with a 40x C-Apochromat (1.2 NA) water-immersion objective and collected the emission onto a Hamamatsu ORCA Flash 4.0 using μManager software. We used BP 440–470 nm to excite GFP and collected BP 525–550 emission using a FT 495 dichroic, and mCherry with BP 530–585 nm excitation and LP 615 emission, using an FT 600 dichroic filter, and mCherry with BP 530–585 nm excitation and LP 615 emission, using an FT 600 dichroic filter. Brightness and contrast are not the same for all images.

For the gametogenesis time-lapse imaging (Fig 1C–1F), we crossed a haploid *Sp* strain carrying *mCherry-wtf4* (SZY1142) to one with *wtf4^poison^-GFP* (SZY5047) to generate heterozygous diploids as previously reported [37]. We also crossed a haploid *Sp* strain carrying *wtf4^antidote^-mCherry* (SZY2572) to one with *wtf4^poison^-GFP* (SZY5047) to generate heterozygous diploids using the same method as above. We grew these diploids to saturation in 5 mLs of rich YEL broth (0.5% yeast extract, 3% glucose, 250 mg/L of adenine, lysine, histidine, leucine, and uracil) overnight at 32°C. We then diluted 100 μL of these diploid cultures into 5 mLs of PM media (20 mLs of 50x EMM salts, 20 ml 0.4 M Na_2_HPO_4_, 25 mL 20% NH_4_Cl, 1 mL 1000x vitamins, 100 μL 10,000x mineral stock solution, 3 g potassium hydrogen phthalate, 950 mL ddH_2_O, 25 mL of sterile 40% glucose after autoclaving, supplemented with 250 mg/L uracil) and again grew overnight at 32°C. The next day, we spun to pellet and resuspended the pellet in PM-N media (PM without NH_4_Cl). We shook the PM-N cultures for 4 h at 28°C. We loaded the plate with PM-N media after washing and flushing the microfluidic plate with PBS. Diploids were trapped in an EMD Millipore CellASIC (Haploid budding yeast) microfluidic plate. PM-N media was flowed through the plate for the duration of the movie. The temperature was maintained through the CellASIC manifold at 25°C. GFP and mCherry fluorescence were excited with a Spectra III illuminator with a 475 nm and 555 nm dichroic, respectively. The fluorescence emission was then collected with a 515/30 nm emission filter for GFP and a 595/31 nm emission filter for mCherry. The fluorescence was recorded by a Prime 95B sCMOS camera (Photometrics). Images from multiple positions were recorded every 10 minutes for 24 hours. Each experiment had two biological replicates of each diploid and was imaged at 4 XY locations analyzed in parallel. We repeated the experiments with both diploids twice. The total number of diploid cells analyzed were n = 25 for *mCherry-wtf4*/*wtf4^poison^-GFP* diploids and n = 13 for *wtf4^antidote^-mcherry/wtf4^poison^-GFP* diploids.

Analysis of these data was performed in Fiji (https://imagej.net/software/fiji/) using a few different plugins written in house (https://research.stowers.org/imagejplugins/). First stage drift was eliminated with “stackregj”. The data was then smoothed with a 1-pixel Gaussian blur. Next, the background was subtracted with a 50-pixel rolling ball. After this, ROIs were drawn by hand around each cell of interest. The average intensity over time was plotted using “create spectrum jru v1”. Time traces for GFP and mCherry for each cell were recorded separately. Next, each time trace was aligned in time manually by marking the first frame (from transmitted light only) that spores started to appear and setting that frame to time zero with “set multi plot offsets jru v1”. Then, all time traces for a particular strain and for GFP and mCherry only were combined into one plot with “combine trajectories jru v1”. These plots were then normalized to the minimum and maximum using “normalize trajectories jru v1”. The traces were then imported to Prism 9 (https://www.graphpad.com/scientific-software/prism/) using the XY graph preset, with the mean and 95% confidence interval plotted against time. The time scale for the plots was recalculated using the frame rate of the time lapse.

For the Fluorescence Recovery After Photobleaching experiments, cells were imaged on a Ti2-E (Nikon) microscope coupled to a CSU-W1 Spinning Disc (Yokogawa). GFP and mCherry were laser excited at 488 nm and 561 nm, respectively, through a 60x (spore FRAP NA 1.4) or 100x (whole ascus FRAP NA 1.45) Plan Apochromat objective. The fluorescence emission of GFP was collected through a 525/36 nm filter, while the emission of mCherry was collected through a 605/25 nm filter. Both signals were collected on a Prime 95B camera (Photometrics).

For the spore and whole ascus FRAP experiments, an initial frame of fluorescence was recorded in each channel. Then, many spores or asci were bleached to background in the same large field of view, using 100% power from the 450nm, 550nm and 640nm laser lines. Once bleaching was completed, the recovery was recorded every 10 minutes for ~6 hours total time (whole FRAP) or every 5 minutes for 4 hours total time (spore FRAP). To generate the recovery curves, using Fiji, the average fluorescence intensity was quantified inside all spores containing GFP signal and all spores containing mCherry signal per frame, separately. Once the curves were obtained, all curves of the same fluorophore were normalized to the min and max intensity values and averaged to yield the final curves.

To quantify the amount of fluorescence intensity inside and outside spores, asci were imaged on an LSM 780 (Zeiss) in photon counting mode with a 40x LD C-Apochromat Objective (NA 1.1). GFP and mCherry were excited at 488 and 561nm, respectively. GFP fluorescence was collected through a 482–553 nm bandpass filter and mCherry fluorescence was collected through a 572–735 nm bandpass filter. Single z slices were collected with transmitted light. Then for each ascus, an ROI was drawn around the entire ascus, and around each of the 4 spores individually (drawn on the transmitted light without input from the fluorescence). This occurred either manually or via Cellpose (https://www.cellpose.org/) with manual editing of the auto generated ROIs. Then the sum-total intensity of the ascus in each channel was measured. Then the sum total intensity of each spore in each channel was also measured. For each channel, the 4 spores’ intensities were added together and divided by the total intensity to yield the fractional spore intensity. This was done for each channel individually. The results of many fractional spore intensities were plotted together.

### Analysis of published Mei4 Chip-Seq data

FASTQ data from [50] was retrieved from the European Nucleotide Archive (ENA), with accession number: ERP001894. Data was trimmed for quality using Trimmomatic [83] and was aligned to *S*. *pombe* genome version ASM294v2 with bowtie2 [84] using default parameters. Peaks were called using MACS2 with callpeak -gsize 1.38^e7^ and -q 0.05. Metagene plots were constructed by averaging the RPM (Reads Per Million) signal for individual genes (either the predicted *wtf* meiotic drive genes (*wtf4*, *wtf13*, top*)* or the *wtf* genes that are predicted to only encode antidote proteins (*wtf5*, *wtf9*, *wtf10*, *wtf16*, *wtf18*, *wtf20*, *wtf21*, *wtf25*, bottom)) across 400 bins and then averaging multiple genes to create a single profile followed by loess smoothing with a span of 0.05. For any reads that mapped to more than one location, only a single location, chosen at random, is reported.

## Supporting information

S1 Fig Representation of alleles used in this study. Each allele is represented as a cartoon with the allele numbers referenced through the text. The depictions describe the promoters (orange for *p*^*antidote*^ and yellow for *p*^*poison*^), the translational start site of the proteins (black arrows), fluorescent tags (mCherry or GFP), translational start site mutations (red stars) and each protein produced. Wtf4^antidote^ is represented as “A” and Wtf4^poison^ is represented as “P”. We also depict the FLEX motif (black box) found within *p*^*poison*^. These depictions are also present in figures where each of these alleles is used.
(TIF)

S2 Fig C-terminal tag reveals the expression of Wtf4^antidote^ prior to spore formation. (**A**) Allele transmission and fertility (assayed via viable spore yield) of 15 diploids with the depicted genotypes. The genotype column shows a cartoon depiction of the relevant genotype. The progeny phenotypes are then shown on the right. For diploids heterozygous at one locus (e.g., Diploid 1), two values are shown (top and bottom) that represent the two possible haploid genotypes. The depictions are not to scale with the location of the loci on the chromosomes. Spores exhibiting both parental phenotypes were considered diploid or aneuploid and were excluded from this table but can be found in S1 Data. The expected values assume Mendelian allele transmission. We used the viable spore yield assay (VSY) to quantify fertility with values normalized to the relevant empty vector control (* = p < 0.05, NS = not significant; G-test for allele transmission, Wilcoxon test for VSY, in comparison to the empty vector control). We compared diploids 3, 4, 5, 6, 8, 10, 12, 13 to control diploid 1 and diploids 7, 9, 11, 14, 15 to control diploid 2. The data for diploids 1, 2, and 12–15 are also depicted in S10A Fig and the data for diploids 1 and 5 are also depicted in Fig 3C. The data from diploids 3, 4, and 14 were previously published in [37]. (**B**) Images of a heterozygous *mCherry-wtf4*^*antidote*^*/ade6+* diploid cell and mature ascus. mCherry-Wtf4^antidote^ is shown in magenta in merged images. (**C**) Images of a heterozygous *wtf4*^*antidote*^*-mCherry/ura4+* diploid cell and mature ascus. Wtf4^antidote^-mCherry is shown in magenta. (**D**) Images of a heterozygous *wtf4*^*antidote*^*-GFP/ade6+* diploid cell and mature ascus. Wtf4^antidote^-GFP is shown in magenta. All Images were taken after 3 days on sporulation media. TL = transmitted light. All scale bars represent 2 μm. Images were taken with the same settings. Not all images are shown at the same brightness and contrast to avoid over saturation of pixels in the brighter images.
(TIF)

S3 Fig Wtf4^poison^ is produced prior to spore individualization. (**A**) Representative images from a Fluorescence Recovery After Photobleaching (FRAP) experiment with mature asci (n = 10) generated from *mCherry-wtf4/ wtf4*^*poison*^-GFP diploids. (**B**) FRAP of both mCherry (magenta line) and GFP (cyan line) post bleaching to 0% intensity and recovery quantified over 6 hours. (**C**) Representative images from a FRAP experiment with spores in mature asci (n = 40) generated from *wtf4*^*antidote*^*-mCherry/wtf4*^*poison*^-GFP diploids. (**D**) FRAP of GFP (cyan line) post bleaching to 0% intensity and recovery was quantified over 3 hours. Not all images are shown at the same brightness and contrast to avoid over saturation of pixels in the brighter images.
(TIF)

S4 Fig Wtf4^poison^ and Wtf4^antidote^ protein expression time-course in *S*. *pombe* meiosis. (**A**) Time course of *wtf4*^*antidote*^*-mCherry*/*wtf4*^*poison*^*-GFP* diploid showing the localization of mCherry-Wtf4^antidote^ (magenta in merged images) and Wtf4^poison^-GFP (cyan in merged images) after the indicated times in PM-N media. All scale bars represent 10 μm. These are images of the cell populations sampled for the westerns. (**B**) Western blot of whole cell extracts from cells expressing *wtf4*^*poison*^*-GFP* at the times indicated. *Indicates a non-specific band. Two biological replicates are shown on the gel. (**C**) Western blot of whole cell extracts from cells expressing *wtf4*^*antidote*^*-mCherry* at the times indicated. (**D**) A replicate gel was stained with Imperial protein stain to show quantity of protein loaded.
(TIF)

S5 Fig Wtf4^antidote^-mCherry and mCherry- Wtf4^antidote^ protein expression time-course in *S*. *pombe* meiosis. (**A**) Time course of *mCherry- wtf4*^*antidote*^/EV *and wtf4*^*antidote*^*-mCherry/*EV diploids showing the localization of Wtf4^antidote^ (magenta in merged images) after the indicated times in PM-N media. All scale bars represent 10 μm. These are images of the cell populations sampled for the westerns. (**B**) Western blot of whole cell extracts from cells expressing either *mCherry- wtf4*^*antidote*^ or *wtf4*^*antidote*^*-mCherry* at the times indicated. The mCh control was isolated from mitotically growing cells expressing constitutive mCherry. (**C**) A replicate gel was stained with Imperial protein stain to show quantity of protein loaded.
(TIF)

S6 Fig Wtf4^poison^ and Wtf4^antidote^ protein expression time-course in synchronized *S*. *pombe* meiosis. **(A)** Time course showing meiotic progression of h-/h- *pat1*.*L95G/pat1*.*L95G* diploid cells that were heterozygous for both *wtf4*^*poison*^*-GFP* and *wtf4*^*antidote*^*-mCherry* [57] 0–24 hours (top) following the addition of 3-MB-PP1 (25 μM) to induce meiosis. The number of nuclei in DAPI-stained cells was counted at the indicated time points. **(B)** Anti-GFP Western blot of whole cell extracts of cells from the same experiment depicted in **A.** Bands consistent with Wtf4^poison^-GFP and free GFP are highlighted with arrows. *indicates a non-specific band. The GFP and mCherry controls were prepared from sporulated cells expressing the free fluorophores constitutively (SZY2636 and SZY2638; see methods). The DMSO control was prepared from a diploid with the same genotype as the experimental sample, but treated for 24 hours with DMSO instead off 3-MB-PP1. **(C)** Anti-mCherry Western blot on the same membrane as that shown in **B**. Bands consistent with Wtf4^antidote^-mCherry and free mCherry are highlighted with arrows. **(D)** Ponceau staining of the membrane in **B-C**. **(E)** The same protein samples used in the blot in **B-C** were rerun on a new anti-mCherry Western where blotting stringency was increased by the addition of 0.2% Tween 20 to the primary antibody. A band consistent with Wtf4^antidote^-mCherry is observed and is not present in the negative control lanes. **(F)** Ponceau staining of the membrane shown in **E**. The DMSO control sample was erroneously not loaded on this blot.
(TIF)

S7 Fig Expression of *wtf4*^*antidote*^ promoter reporters in diploids and asci. Depictions and images of the two lengths of *wtf4*^*antidote*^ promoters used in this study, *p*^*antidote*^ (**A**) and *p*^*antidote long*^ (**B**). The images of the two different *p*^*antidote*^-*mCherry* reporters */ +* were from heterozygous diploids and asci. (**C**) Quantification of mCherry fluorescence within heterozygous (*p*^*antidote*^-*mCherry* reporter*/ +*) diploids and asci. At least 25 diploids and 25 asci were quantified per reporter. All images were acquired after 3 days on sporulation media. TL = transmitted light. All scale bars represent 2 μm. Images were taken at the same settings and are shown at the same brightness and contrast for accurate comparison.
(TIF)

S8 Fig Mei4 associates with LTRs in meiosis. Peaks (called by MACS2) from Mei4 ChIP-seq (data from [50]) 4 hours past meiotic induction are compared to LTR regions (identified by BLAST). If the regions overlap, they are shown in the shared region of the Venn diagram. For any ChIP-seq reads that mapped to more than one location, only a single location, chosen at random, was selected.
(TIF)

S9 Fig Wtf4^poison^ and Wtf4^antidote^ functions are disrupted when expressed from the p^antidote^ and p^poison^ promoter, respectively. (**A**) Allele transmission and fertility (assayed via viable spore yield) of 11 diploids of the depicted genotypes. The genotype column shows a cartoon depiction of the relevant genotype. The progeny phenotypes are then shown on the right. For diploids heterozygous at one locus (e.g., diploid 1), two values are shown (top and bottom) that represent the two possible haploid genotypes. For diploids heterozygous at two loci, the loci used are unlinked and should segregate randomly. The depictions are not to scale with the location of the loci on the chromosomes. Spores exhibiting both parental phenotypes were considered diploid or aneuploid and were excluded from this table but can be found in S1 Data. The expected values assume Mendelian allele transmission. (* = p < 0.05, NS = not significant; G-test for allele transmission, Wilcoxon test for VSY, in comparison to the empty vector control). We compared diploids 12, 13, 14, 20 to diploid 1 as the control and diploids 15, 21, 22, 23, 24 to diploid 2 as the control. The data for the control diploids 1–2 and 12–15 are also depicted in S2A Fig. The data for diploid 1 is also in Fig 3C. (**B**) Images of *p*^*antidote*^-*wtf4*^*poison*^*-GFP/ade6+*, *wtf4*^*antidote*^*-mCherry/ura4+* parental and non-parental ditype asci. p^antidote^-Wtf4^poison^-GFP is shown in cyan and Wtf4^antidote^-mCherry is shown in magenta in merged images. TL = transmitted light. All scale bars represent 2 μm. All images acquired after 2 days on sporulation media. All images are shown at the same brightness and contrast for accurate comparison.
(TIF)

S1 Video Time-lapse videos of representative cells shown in Fig 1C and 1E. Panel A in the video shows a representative *mCherry-wtf4/wtf4*^*poison*^*-GFP* diploid undergoing meiosis, captured by the time lapse microscopy we conducted in Fig 1C. mCherry-Wtf4^antidote^ is in magenta and Wtf4^poison^-GFP is in cyan in the composite. Panel B in the video shows a representative *wtf4*^*antidote*^*-mcherry/wtf4*^*poison*^*-GFP* diploid undergoing meiosis, captured by the time lapse microscopy we conducted in Fig 1E. Wtf4^antidote^-mCherry is in magenta and Wtf4^poison^-GFP is in cyan in the composite. Scale bar represents 4 μm. The videos were captured using the same settings. The videos are not shown at the same brightness and contrast to avoid over saturation of pixels in the brighter frames. Each video is comprised of 115 frames captured at 10 minute intervals.
(MOV)

S1 Table Yeast strains used. Column 1 is the name of strain used, while column 2 refers to the genotype. Columns 3 lists the reference for the yeast strain. If it was made in this study, we also detail how the strain was made in column 4. Column 5 lists the figure(s) in which the strain was used.
(XLSX)

S2 Table Plasmids used. Column 1 is the name of plasmid used. Column 2 gives a short description of the plasmid. Columns 3 lists the reference for the plasmid.
(XLSX)

S3 Table Oligos Used. Column 1 is the name of oligo used. Column 2 details the sequence of the oligo, while column 3 gives a short description.
(XLSX)

S1 Data Genetics and Viable Spore Yield (VSY) data for all diploids presented. Tab 1 is a summary of all the allele transmission and VSY data for all 22 diploids. Each subsequent tab contains the raw data for individual crosses. The tab name contains the diploid number, corresponding to the diploid number in the paper, and the two strains crossed to generate the diploid. The top table in each tab lists the VSY data, including the data for at least 3 independent diploids (A-X) per cross. The number of colonies for the diploid dilutions and the corresponding spore dilutions are listed, as well as the average viable spore yield, standard deviation, and the p-value when compared to the appropriate control (Wilcoxon test, * = p < 0.05, NS = not significant). The bottom table contains the allele transmission data. We detail the genotypes of the two strains crossed to generate the diploid (allele 1 and allele 2) and the transmission frequencies of the two alleles, as well as a control locus. We show the data with and without disomes in the table, but used the data excluding disomes in the figures. We also list the p-values compared to appropriate control (G-test, * = p < 0.05, NS = not significant).
(XLSX)

S2 Data Raw data of image quantification. Each tab contains the raw data of quantifications presented in the manuscript. The first tab contains the quantifications depicted in Fig 1D and 1F, where the normalized fluorescence intensity of mCherry and GFP were calculated for diploids undergoing meiosis. Traces for each diploid were plotted as the mean with 95% confidence interval against time. The second tab contains the quantification depicted in Fig 2C and 2F of reporter (promoter-FP) expression in spores. For each ascus, we detail the mean fluorescence intensity of the 4 individual spores and the total of the four spores. We then divided each individual spore by the total to get the percent of total spore fluorescence inside a given spore. The third tab details the quantification depicted in Fig 4D of percent intensity inside spores of given fluorescent proteins. We show the percent of fluorescent protein inside spores in three different genotypes (*p*^*poison*^*-wtf4*^*antidote*^*-GFP/mCh-wtf4*^*poison*^, *p*^*poison*^*-wtf4*^*antidote*^*-GFP/ade6+*, *mCh-wtf4*^*poison*^*/ade6+)*. The fourth tab details the Fluorescence Recovery After Photobleaching (FRAP) quantifications depicted in S3B Fig. We normalized the fluorescence intensity before bleaching to 1. Once bleaching was completed, the recovery was recorded every 10 minutes for approximately 6 hours. The fluorescence levels of both mCherry-Wtf4 and Wtf4^poison^-GFP are recorded for each time point. The fifth tab details the Fluorescence Recovery After Photobleaching (FRAP) quantifications depicted in S3D Fig. The sixth tab details the quantifications shown in S7C Fig, with the fluorescence intensity inside asci and diploids for the following constructs: p^antidote^-mCherry, p^antidote long^-mCherry, and the empty vector control. The seventh tab details Fig 5D, the percent intensity inside spores of given fluorescent proteins. We show the percent of Wtf4^poison^-GFP inside spores in three different genotypes: *p*^*antidote*^*-wtf4*^*poison*^*-GFP/ade6+*, *wtf4*^*antidote*^*-mCh/ura4+; wtf4*^*poison*^*-GFP/ade6+*; and *wtf4*^*poison*^*-GFP/ade6+*, *wtf4*^*antidote-*^*mCh/ura4+*.
(XLSX)

## References

[1] Abbott S, Fairbanks DJ. Experiments on Plant Hybrids by Gregor Mendel. Genetics. 2016;204(2):407–22. doi: 10.1534/genetics.116.195198 27729492 PMC5068836

[2] Burt A, Trivers R. Genes in conflict: the biology of selfish genetic elements. Cambridge, Mass.: Belknap Press of Harvard University Press; 2006. viii, 602 p., 8 p. of plates p.

[3] Lindholm AK, Dyer KA, Firman RC, Fishman L, Forstmeier W, Holman L, et al. The Ecology and Evolutionary Dynamics of Meiotic Drive. Trends Ecol Evol. 2016;31(4):315–26. doi: 10.1016/j.tree.2016.02.001 26920473

[4] Akera T, Chmatal L, Trimm E, Yang K, Aonbangkhen C, Chenoweth DM, et al. Spindle asymmetry drives non-Mendelian chromosome segregation. Science. 2017;358(6363):668–72. doi: 10.1126/science.aan0092 29097549 PMC5906099

[5] Bauer H, Schindler S, Charron Y, Willert J, Kusecek B, Herrmann BG. The nucleoside diphosphate kinase gene Nme3 acts as quantitative trait locus promoting non-Mendelian inheritance. PLoS Genet. 2012;8(3):e1002567. doi: 10.1371/journal.pgen.1002567 22438820 PMC3305403

[6] Bauer H, Veron N, Willert J, Herrmann BG. The t-complex-encoded guanine nucleotide exchange factor Fgd2 reveals that two opposing signaling pathways promote transmission ratio distortion in the mouse. Genes Dev. 2007;21(2):143–7. doi: 10.1101/gad.414807 17234881 PMC1770897

[7] Chen J, Ding J, Ouyang Y, Du H, Yang J, Cheng K, et al. A triallelic system of S5 is a major regulator of the reproductive barrier and compatibility of indica-japonica hybrids in rice. Proc Natl Acad Sci U S A. 2008;105(32):11436–41. doi: 10.1073/pnas.0804761105 18678896 PMC2516230

[8] Dalstra HJ, Swart K, Debets AJ, Saupe SJ, Hoekstra RF. Sexual transmission of the [Het-S] prion leads to meiotic drive in *Podospora anserina*. Proc Natl Acad Sci U S A. 2003;100(11):6616–21. doi: 10.1073/pnas.1030058100 12719532 PMC164496

[9] Dawe RK, Lowry EG, Gent JI, Stitzer MC, Swentowsky KW, Higgins DM, et al. A Kinesin-14 Motor Activates Neocentromeres to Promote Meiotic Drive in Maize. Cell. 2018;173(4):839–50 e18. doi: 10.1016/j.cell.2018.03.009 29628142

[10] Didion JP, Morgan AP, Clayshulte AM, McMullan RC, Yadgary L, Petkov PM, et al. A multi-megabase copy number gain causes maternal transmission ratio distortion on mouse chromosome 2. PLoS Genet. 2015;11(2):e1004850. doi: 10.1371/journal.pgen.1004850 25679959 PMC4334553

[11] Grognet P, Lalucque H, Malagnac F, Silar P. Genes that bias Mendelian segregation. PLoS Genet. 2014;10(5):e1004387. doi: 10.1371/journal.pgen.1004387 24830502 PMC4022471

[12] Hammond TM, Rehard DG, Xiao H, Shiu PK. Molecular dissection of *Neurospora* Spore killer meiotic drive elements. Proc Natl Acad Sci U S A. 2012;109(30):12093–8. doi: 10.1073/pnas.1203267109 22753473 PMC3409728

[13] Helleu Q, Gerard PR, Dubruille R, Ogereau D, Prud’homme B, Loppin B, et al. Rapid evolution of a Y-chromosome heterochromatin protein underlies sex chromosome meiotic drive. Proc Natl Acad Sci U S A. 2016;113(15):4110–5. doi: 10.1073/pnas.1519332113 26979956 PMC4839453

[14] Herrmann BG, Koschorz B, Wertz K, McLaughlin KJ, Kispert A. A protein kinase encoded by the t complex responder gene causes non-mendelian inheritance. Nature. 1999;402(6758):141–6. doi: 10.1038/45970 10647005

[15] Kruger AN, Brogley MA, Huizinga JL, Kidd JM, de Rooij DG, Hu YC, et al. A Neofunctionalized X-Linked Ampliconic Gene Family Is Essential for Male Fertility and Equal Sex Ratio in Mice. Curr Biol. 2019;29(21):3699–706.e5. doi: 10.1016/j.cub.2019.08.057 31630956 PMC7012382

[16] Larracuente AM, Presgraves DC. The selfish *Segregation Distorter* gene complex of *Drosophila melanogaster*. Genetics. 2012;192(1):33–53. doi: 10.1534/genetics.112.141390 22964836 PMC3430544

[17] Lin CJ, Hu F, Dubruille R, Vedanayagam J, Wen J, Smibert P, et al. The hpRNA/RNAi Pathway Is Essential to Resolve Intragenomic Conflict in the *Drosophila* Male Germline. Dev Cell. 2018;46(3):316–26 e5. doi: 10.1016/j.devcel.2018.07.004 30086302 PMC6114144

[18] Long Y, Zhao L, Niu B, Su J, Wu H, Chen Y, et al. Hybrid male sterility in rice controlled by interaction between divergent alleles of two adjacent genes. Proc Natl Acad Sci U S A. 2008;105(48):18871–6. doi: 10.1073/pnas.0810108105 19033192 PMC2596266

[19] Phadnis N, Orr HA. A single gene causes both male sterility and segregation distortion in *Drosophila* hybrids. Science. 2009;323(5912):376–9. doi: 10.1126/science.1163934 19074311 PMC2628965

[20] Pieper KE, Unckless RL, Dyer KA. A fast-evolving X-linked duplicate of importin-α2 is overexpressed in sex-ratio drive in *Drosophila neotestacea*. Mol Ecol. 2018;27(24):5165–79. 30411843 10.1111/mec.14928 PMC6312747

[21] Rathje CC, Johnson EEP, Drage D, Patinioti C, Silvestri G, Affara NA, et al. Differential Sperm Motility Mediates the Sex Ratio Drive Shaping Mouse Sex Chromosome Evolution. Curr Biol. 2019;29(21):3692–8.e4. doi: 10.1016/j.cub.2019.09.031 31630954 PMC6839398

[22] Rhoades NA, Harvey AM, Samarajeewa DA, Svedberg J, Yusifov A, Abusharekh A, et al. Identification of *rfk-1*, a Meiotic Driver Undergoing RNA Editing in *Neurospora*. Genetics. 2019;212(1):93–110. doi: 10.1534/genetics.119.302122 30918007 PMC6499513

[23] Shen R, Wang L, Liu X, Wu J, Jin W, Zhao X, et al. Genomic structural variation-mediated allelic suppression causes hybrid male sterility in rice. Nat Commun. 2017;8(1):1310. doi: 10.1038/s41467-017-01400-y 29101356 PMC5670240

[24] Svedberg J, Vogan AA, Rhoades NA, Sarmarajeewa D, Jacobson DJ, Lascoux M, et al. An introgressed gene causes meiotic drive in *Neurospora sitophila*. Proc Natl Acad Sci U S A. 2021;118(17). doi: 10.1073/pnas.2026605118 33875604 PMC8092558

[25] Vogan AA, Ament-Velasquez SL, Granger-Farbos A, Svedberg J, Bastiaans E, Debets AJ, et al. Combinations of *Spok* genes create multiple meiotic drivers in *Podospora*. Elife. 2019;8. doi: 10.7554/eLife.46454 31347500 PMC6660238

[26] Wu CI, Lyttle TW, Wu ML, Lin GF. Association between a satellite DNA sequence and the Responder of *Segregation Distorter* in *D. melanogaster*. Cell. 1988;54(2):179–89. doi: 10.1016/0092-8674(88)90550-8 2839299

[27] Xie Y, Tang J, Xie X, Li X, Huang J, Fei Y, et al. An asymmetric allelic interaction drives allele transmission bias in interspecific rice hybrids. Nat Commun. 2019;10(1):2501. doi: 10.1038/s41467-019-10488-3 31175302 PMC6555797

[28] Yu X, Zhao Z, Zheng X, Zhou J, Kong W, Wang P, et al. A selfish genetic element confers non-Mendelian inheritance in rice. Science. 2018;360(6393):1130–2. doi: 10.1126/science.aar4279 29880691

[29] Sandler L, Novitski E. Meiotic Drive as an Evolutionary Force. The American Naturalist. 1957;91(857):105–10.

[30] Johnson NA. Hybrid incompatibility genes: remnants of a genomic battlefield? Trends Genet. 2010;26(7):317–25. doi: 10.1016/j.tig.2010.04.005 20621759

[31] Presgraves DC. The molecular evolutionary basis of species formation. Nat Rev Genet. 2010;11(3):175–80. doi: 10.1038/nrg2718 20051985

[32] Segurel L, Leffler EM, Przeworski M. The case of the fickle fingers: how the PRDM9 zinc finger protein specifies meiotic recombination hotspots in humans. PLoS Biol. 2011;9(12):e1001211. doi: 10.1371/journal.pbio.1001211 22162947 PMC3232208

[33] Zanders SE, Unckless RL. Fertility Costs of Meiotic Drivers. Curr Biol. 2019;29(11):R512–R20. 31163165 10.1016/j.cub.2019.03.046 PMC6586414

[34] Bravo Nunez MA, Nuckolls NL, Zanders SE. Genetic Villains: Killer Meiotic Drivers. Trends Genet. 2018;34(6):424–33. 29499907 10.1016/j.tig.2018.02.003 PMC5959745

[35] Bravo Nunez MA, Sabbarini IM, Eickbush MT, Liang Y, Lange JJ, Kent AM, et al. Dramatically diverse *Schizosaccharomyces* pombe wtf meiotic drivers all display high gamete-killing efficiency. PLoS Genet. 2020;16(2):e1008350. doi: 10.1371/journal.pgen.1008350 32032353 PMC7032740

[36] Eickbush MT, Young JM, Zanders SE. Killer Meiotic Drive and Dynamic Evolution of the *wtf* Gene Family. Mol Biol Evol. 2019;36(6):1201–14. doi: 10.1093/molbev/msz052 30991417 PMC6526906

[37] Nuckolls NL, Bravo Nunez MA, Eickbush MT, Young JM, Lange JJ, Yu JS, et al. *wtf* genes are prolific dual poison-antidote meiotic drivers. Elife. 2017;6. doi: 10.7554/eLife.26033 28631612 PMC5478261

[38] Hu W, Jiang ZD, Suo F, Zheng JX, He WZ, Du LL. A large gene family in fission yeast encodes spore killers that subvert Mendel’s law. Elife. 2017;6. doi: 10.7554/eLife.26057 28631610 PMC5478263

[39] Bravo Nunez MA, Lange JJ, Zanders SE. A suppressor of a *wtf* poison-antidote meiotic driver acts via mimicry of the driver’s antidote. PLoS Genet. 2018;14(11):e1007836. doi: 10.1371/journal.pgen.1007836 30475921 PMC6283613

[40] Avelar AT, Perfeito L, Gordo I, Ferreira MG. Genome architecture is a selectable trait that can be maintained by antagonistic pleiotropy. Nat Commun. 2013;4:2235. doi: 10.1038/ncomms3235 23974178

[41] Bravo Nunez MA, Sabbarini IM, Eide LE, Unckless RL, Zanders SE. Atypical meiosis can be adaptive in outcrossed *Schizosaccharomyces pombe* due to *wtf* meiotic drivers. Elife. 2020;9. doi: 10.7554/eLife.57936 32790622 PMC7426094

[42] Hu W, Suo F, Du LL. Bulk Segregant Analysis Reveals the Genetic Basis of a Natural Trait Variation in Fission Yeast. Genome Biol Evol. 2015;7(12):3496–510. doi: 10.1093/gbe/evv238 26615217 PMC4700965

[43] Jeffares DC, Jolly C, Hoti M, Speed D, Shaw L, Rallis C, et al. Transient structural variations have strong effects on quantitative traits and reproductive isolation in fission yeast. Nat Commun. 2017;8:14061. doi: 10.1038/ncomms14061 28117401 PMC5286201

[44] Singh G, Klar AJ. The 2.1-kb inverted repeat DNA sequences flank the *mat2,3* silent region in two species of *Schizosaccharomyces* and are involved in epigenetic silencing in Schizosaccharomyces pombe. Genetics. 2002;162(2):591–602. doi: 10.1093/genetics/162.2.591 12399374 PMC1462298

[45] Zanders SE, Eickbush MT, Yu JS, Kang JW, Fowler KR, Smith GR, et al. Genome rearrangements and pervasive meiotic drive cause hybrid infertility in fission yeast. Elife. 2014;3:e02630. doi: 10.7554/eLife.02630 24963140 PMC4066438

[46] Hansen KR, Burns G, Mata J, Volpe TA, Martienssen RA, Bähler J, et al. Global effects on gene expression in fission yeast by silencing and RNA interference machineries. Mol Cell Biol. 2005;25(2):590–601. doi: 10.1128/MCB.25.2.590-601.2005 15632061 PMC543407

[47] Nicolas E, Yamada T, Cam HP, Fitzgerald PC, Kobayashi R, Grewal SI. Distinct roles of HDAC complexes in promoter silencing, antisense suppression and DNA damage protection. Nat Struct Mol Biol. 2007;14(5):372–80. doi: 10.1038/nsmb1239 17450151

[48] Watts BR, Wittmann S, Wery M, Gautier C, Kus K, Birot A, et al. Histone deacetylation promotes transcriptional silencing at facultative heterochromatin. Nucleic Acids Res. 2018;46(11):5426–40. doi: 10.1093/nar/gky232 29618061 PMC6009587

[49] Wei Y, Lee NN, Pan L, Dhakshnamoorthy J, Sun LL, Zofall M, et al. TOR targets an RNA processing network to regulate facultative heterochromatin, developmental gene expression and cell proliferation. Nat Cell Biol. 2021;23(3):243–56. doi: 10.1038/s41556-021-00631-y 33574613 PMC9260697

[50] Alves-Rodrigues I, Ferreira PG, Moldón A, Vivancos AP, Hidalgo E, Guigó R, et al. Spatiotemporal Control of Forkhead Binding to DNA Regulates the Meiotic Gene Expression Program. Cell Rep. 2016;14(4):885–95. doi: 10.1016/j.celrep.2015.12.074 26804917

[51] Ioannoni R, Brault A, Labbé S. Cuf2 Is a Transcriptional Co-Regulator that Interacts with Mei4 for Timely Expression of Middle-Phase Meiotic Genes. PLOS ONE. 2016;11(3):e0151914. doi: 10.1371/journal.pone.0151914 26986212 PMC4795683

[52] Nuckolls NL, Mok AC, Lange JJ, Yi K, Kandola TS, Hunn AM, et al. The *wtf4* meiotic driver utilizes controlled protein aggregation to generate selective cell death. Elife. 2020;9. doi: 10.7554/eLife.55694 33108274 PMC7591262

[53] Smith GR. Genetic analysis of meiotic recombination in *Schizosaccharomyces pombe*. Methods Mol Biol. 2009;557:65–76. doi: 10.1007/978-1-59745-527-5_6 19799177 PMC2758532

[54] Badrinarayanan A, Reyes-Lamothe R, Uphoff S, Leake MC, Sherratt DJ. In vivo architecture and action of bacterial structural maintenance of chromosome proteins. Science. 2012;338(6106):528–31. doi: 10.1126/science.1227126 23112333 PMC3807729

[55] Khmelinskii A, Keller PJ, Bartosik A, Meurer M, Barry JD, Mardin BR, et al. Tandem fluorescent protein timers for in vivo analysis of protein dynamics. Nature Biotechnology. 2012;30(7):708–14. doi: 10.1038/nbt.2281 22729030

[56] Shashkova S, Wollman AJ, Hohmann S, Leake MC. Characterising Maturation of GFP and mCherry of Genomically Integrated Fusions in *Saccharomyces cerevisiae*. Bio Protoc. 2018;8(2):e2710. doi: 10.21769/BioProtoc.2710 29430484 PMC5802385

[57] Guerra-Moreno A, Alves-Rodrigues I, Hidalgo E, Ayté J. Chemical genetic induction of meiosis in *Schizosaccharomyces pombe*. Cell Cycle. 2012;11(8):1621–5. doi: 10.4161/cc.20051 22456336

[58] Antúnez de Mayolo A, Lisby M, Erdeniz N, Thybo T, Mortensen UH, Rothstein R. Multiple start codons and phosphorylation result in discrete Rad52 protein species. Nucleic Acids Res. 2006;34(9):2587–97. doi: 10.1093/nar/gkl280 16707661 PMC1463902

[59] Kuang Z, Boeke JD, Canzar S. The dynamic landscape of fission yeast meiosis alternative-splice isoforms. Genome Res. 2017;27(1):145–56. doi: 10.1101/gr.208041.116 27856494 PMC5204338

[60] Bowen NJ, Jordan IK, Epstein JA, Wood V, Levin HL. Retrotransposons and their recognition of pol II promoters: a comprehensive survey of the transposable elements from the complete genome sequence of *Schizosaccharomyces pombe*. Genome Res. 2003;13(9):1984–97. doi: 10.1101/gr.1191603 12952871 PMC403668

[61] Mata J, Wilbrey A, Bähler J. Transcriptional regulatory network for sexual differentiation in fission yeast. Genome Biology. 2007;8(10):R217. doi: 10.1186/gb-2007-8-10-r217 17927811 PMC2246291

[62] Horie S, Watanabe Y, Tanaka K, Nishiwaki S, Fujioka H, Abe H, et al. The *Schizosaccharomyces pombe mei4*+ gene encodes a meiosis-specific transcription factor containing a forkhead DNA-binding domain. Mol Cell Biol. 1998;18(4):2118–29. doi: 10.1128/MCB.18.4.2118 9528784 PMC121445

[63] Moldón A, Malapeira J, Gabrielli N, Gogol M, Gómez-Escoda B, Ivanova T, et al. Promoter-driven splicing regulation in fission yeast. Nature. 2008;455(7215):997–1000. doi: 10.1038/nature07325 18815595

[64] De Carvalho M, Jia GS, Nidamangala Srinivasa A, Billmyre RB, Xu YH, Lange JJ, et al. The *wtf* meiotic driver gene family has unexpectedly persisted for over 100 million years. Elife. 2022;11. doi: 10.7554/eLife.81149 36227631 PMC9562144

[65] Gietz RD, Schiestl RH, Willems AR, Woods RA. Studies on the transformation of intact yeast cells by the LiAc/SS-DNA/PEG procedure. Yeast. 1995;11(4):355–60. doi: 10.1002/yea.320110408 7785336

[66] Murray JM, Watson AT, Carr AM. Transformation of *Schizosaccharomyces pombe*: Electroporation Procedure. Cold Spring Harb Protoc. 2016;2016(4):pdb.prot090951. doi: 10.1101/pdb.prot090951 27037074

[67] Neiman AM. Sporulation in the budding yeast *Saccharomyces cerevisiae*. Genetics. 2011;189(3):737–65. doi: 10.1534/genetics.111.127126 22084423 PMC3213374

[68] Gütz H, Doe FJ. On Homo- and Heterothallism in *Schizosaccharomyces pombe*. Mycologia. 1975;67(4):748–59. 1177967

[69] Tao YT, Suo F, Tusso S, Wang YK, Huang S, Wolf JBW, et al. Intraspecific Diversity of Fission Yeast Mitochondrial Genomes. Genome Biol Evol. 2019;11(8):2312–29. doi: 10.1093/gbe/evz165 31364709 PMC6736045

[70] Crow JF. Why is Mendelian segregation so exact? Bioessays. 1991;13(6):305–12. doi: 10.1002/bies.950130609 1909864

[71] Zilio N, Codlin S, Vashisht AA, Bitton DA, Head SR, Wohlschlegel JA, et al. A novel histone deacetylase complex in the control of transcription and genome stability. Mol Cell Biol. 2014;34(18):3500–14. doi: 10.1128/MCB.00519-14 25002536 PMC4135615

[72] Sugiyama T, Sugioka-Sugiyama R. Red1 promotes the elimination of meiosis-specific mRNAs in vegetatively growing fission yeast. Embo j. 2011;30(6):1027–39. doi: 10.1038/emboj.2011.32 21317872 PMC3061036

[73] Ioannoni R, Beaudoin J, Lopez-Maury L, Codlin S, Bahler J, Labbe S. Cuf2 Is a Novel Meiosis-Specific Regulatory Factor of Meiosis Maturation. PLOS ONE. 2012;7(4):e36338. doi: 10.1371/journal.pone.0036338 22558440 PMC3338643

[74] Abe H, Shimoda C. Autoregulated expression of *Schizosaccharomyces pombe* meiosis-specific transcription factor Mei4 and a genome-wide search for its target genes. Genetics. 2000;154(4):1497–508. doi: 10.1093/genetics/154.4.1497 10747048 PMC1461037

[75] Murakami-Tonami Y, Yamada-Namikawa C, Tochigi A, Hasegawa N, Kojima H, Kunimatsu M, et al. Mei4p coordinates the onset of meiosis I by regulating *cdc25+* in fission yeast. Proc Natl Acad Sci U S A. 2007;104(37):14688–93. doi: 10.1073/pnas.0702906104 17804800 PMC1976235

[76] Shimoda C, Hirata A, Kishida M, Hashida T, Tanaka K. Characterization of meiosis-deficient mutants by electron microscopy and mapping of four essential genes in the fission yeast *Schizosaccharomyces pombe*. Mol Gen Genet. 1985;200(2):252–7. doi: 10.1007/BF00425432 3861929

[77] Ruan K, Yamamoto TG, Asakawa H, Chikashige Y, Masukata H, Haraguchi T, et al. Meiotic nuclear movements in fission yeast are regulated by the transcription factor Mei4 downstream of a Cds1-dependent replication checkpoint pathway. Genes Cells. 2015;20(3):160–72. doi: 10.1111/gtc.12207 25492408 PMC4359684

[78] Lopez Hernandez JF, Helston RM, Lange JJ, Billmyre RB, Schaffner SH, Eickbush MT, et al. Diverse mating phenotypes impact the spread of *wtf* meiotic drivers in *Schizosaccharomyces pombe*. Elife. 2021;10. doi: 10.7554/eLife.70812 34895466 PMC8789285

[79] Hailey DW, Davis TN, Muller EG. Fluorescence resonance energy transfer using color variants of green fluorescent protein. Methods Enzymol. 2002;351:34–49. doi: 10.1016/s0076-6879(02)51840-1 12073355

[80] Sheff MA, Thorn KS. Optimized cassettes for fluorescent protein tagging in *Saccharomyces cerevisiae*. Yeast. 2004;21(8):661–70. doi: 10.1002/yea.1130 15197731

[81] Flor-Parra I, Zhurinsky J, Bernal M, Gallardo P, Daga RR. A Lallzyme MMX-based rapid method for fission yeast protoplast preparation. Yeast. 2014;31(2):61–6. doi: 10.1002/yea.2994 24323433

[82] Dong Q, Li F. Antibody Pull-Down Experiments in Fission Yeast. Methods Mol Biol. 2018;1721:117–23. 29423852 10.1007/978-1-4939-7546-4_11 PMC6339568

[83] Bolger AM, Lohse M, Usadel B. Trimmomatic: a flexible trimmer for Illumina sequence data. Bioinformatics. 2014;30(15):2114–20. doi: 10.1093/bioinformatics/btu170 24695404 PMC4103590

[84] Langmead B, Salzberg SL. Fast gapped-read alignment with Bowtie 2. Nat Methods. 2012;9(4):357–9. doi: 10.1038/nmeth.1923 22388286 PMC3322381

[85] Lock A, Rutherford K, Harris MA, Hayles J, Oliver SG, Bahler J, et al. PomBase 2018: user-driven reimplementation of the fission yeast database provides rapid and intuitive access to diverse, interconnected information. Nucleic Acids Res. 2018. 10.1093/nar/gky961 PMC6324063 30321395

