# Supplementary figures and images for: S. pombe wtf drivers use dual transcriptional regulation and selective protein exclusion from spores to cause meiotic drive
Source: PLoS Genet. 2022 Dec 7;18(12):e1009847. doi: 10.1371/journal.pgen.1009847 (PMC9762604; doi:10.1371/journal.pgen.1009847)

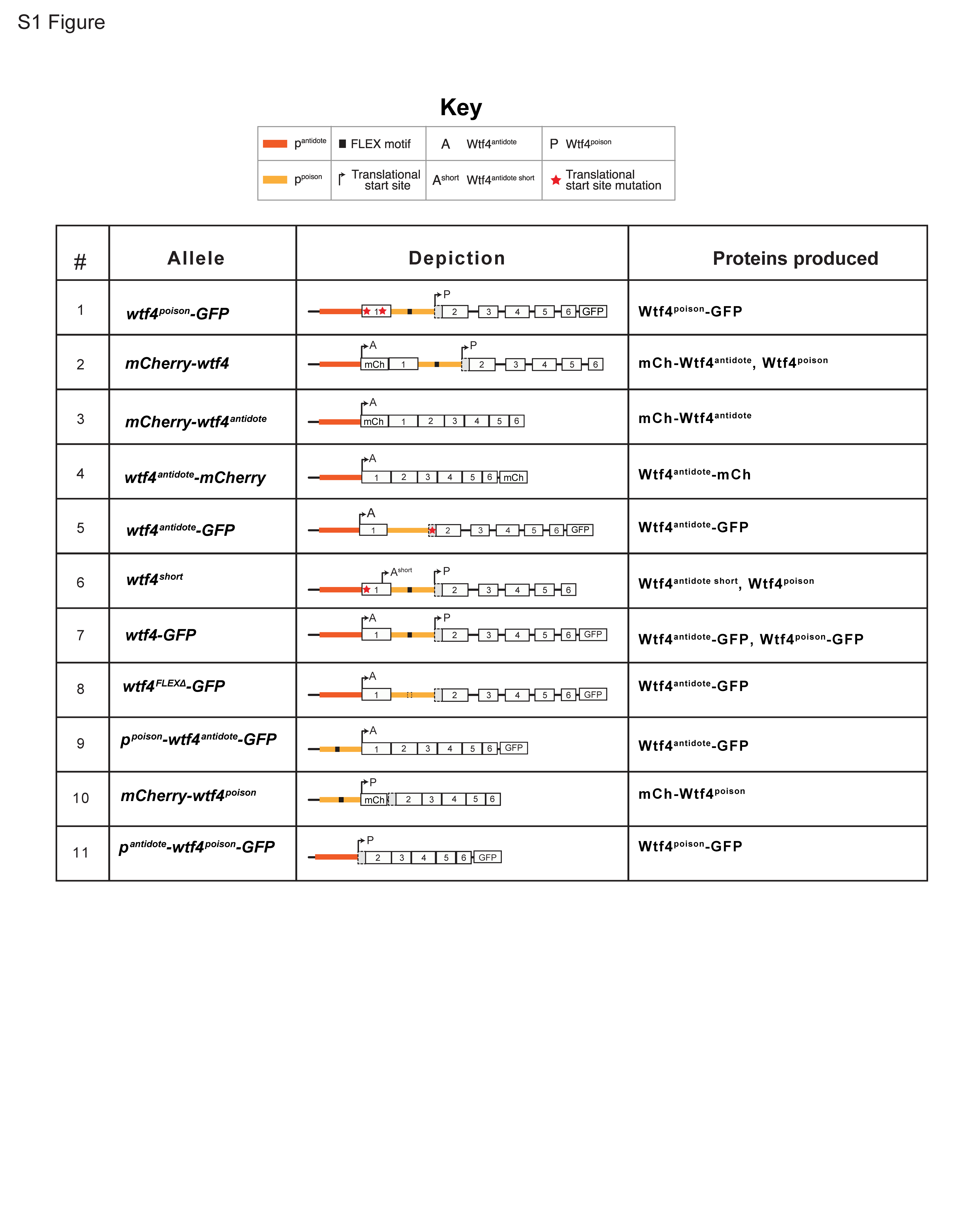

Supplement: S1 Fig — Each allele is represented as a cartoon with the allele numbers referenced through the text. The depictions describe the promoters (orange for pantidote and yellow for ppoison), the translational start site of the proteins (black arrows), fluorescent tags (mCherry or GFP), translational start site mutations (red stars) and each protein produced. Wtf4antidote is represented as “A” and Wtf4poison is represented as “P”. We also depict the FLEX motif (black box) found within ppoison. These depictions are also present in figures where each of these alleles is used. (TIF) [file pgen.1009847.s001.tif]

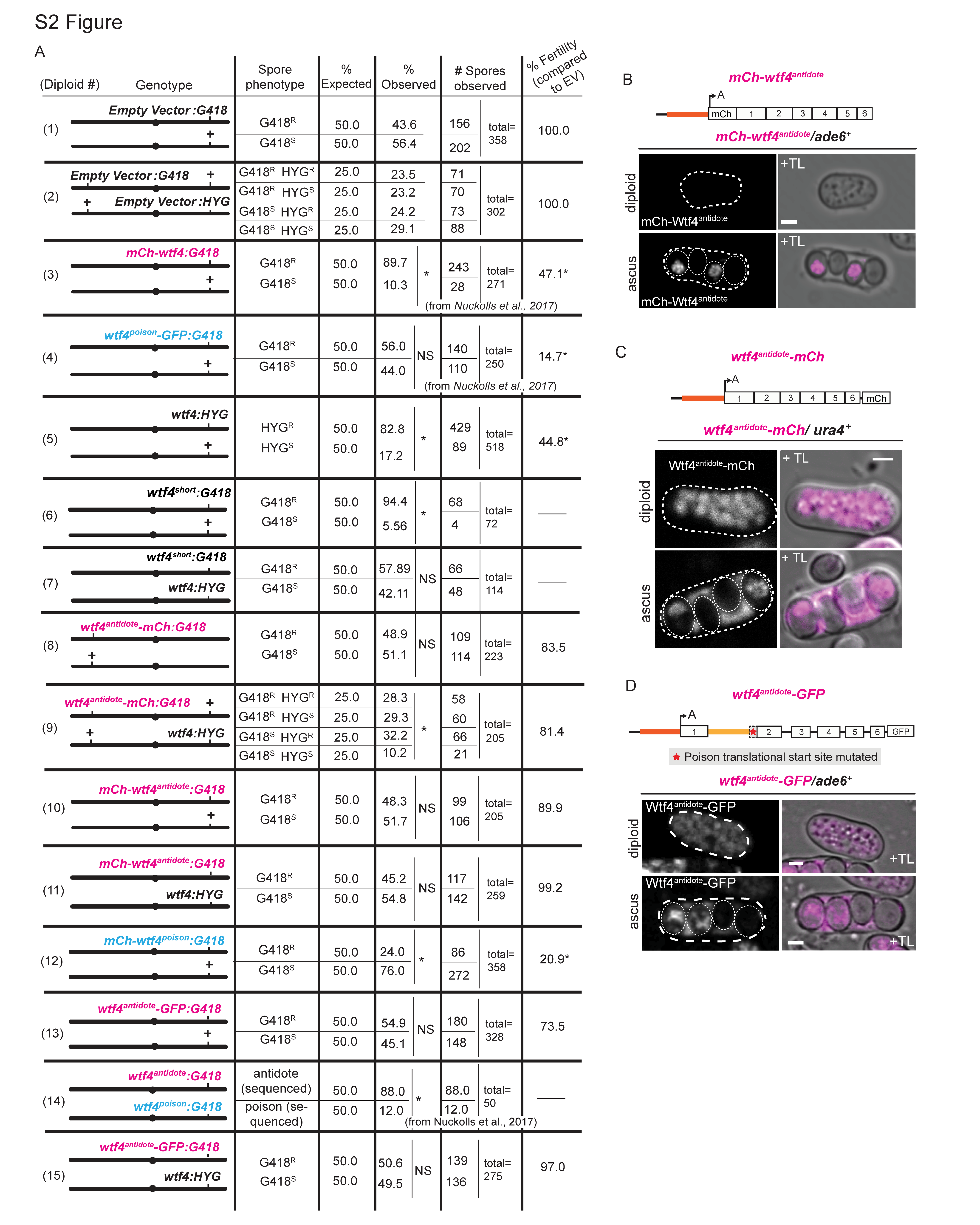

Supplement: S2 Fig — (A) Allele transmission and fertility (assayed via viable spore yield) of 15 diploids with the depicted genotypes. The genotype column shows a cartoon depiction of the relevant genotype. The progeny phenotypes are then shown on the right. For diploids heterozygous at one locus (e.g., Diploid 1), two values are shown (top and bottom) that represent the two possible haploid genotypes. The depictions are not to scale with the location of the loci on the chromosomes. Spores exhibiting both parental phenotypes were considered diploid or aneuploid and were excluded from this table but can be found in S1 Data. The expected values assume Mendelian allele transmission. We used the viable spore yield assay (VSY) to quantify fertility with values normalized to the relevant empty vector control (* = p < 0.05, NS = not significant; G-test for allele transmission, Wilcoxon test for VSY, in comparison to the empty vector control). We compared diploids 3, 4, 5, 6, 8, 10, 12, 13 to control diploid 1 and diploids 7, 9, 11, 14, 15 to control diploid 2. The data for diploids 1, 2, and 12–15 are also depicted in S10A Fig and the data for diploids 1 and 5 are also depicted in Fig 3C. The data from diploids 3, 4, and 14 were previously published in [37]. (B) Images of a heterozygous mCherry-wtf4antidote/ade6+ diploid cell and mature ascus. mCherry-Wtf4antidote is shown in magenta in merged images. (C) Images of a heterozygous wtf4antidote-mCherry/ura4+ diploid cell and mature ascus. Wtf4antidote-mCherry is shown in magenta. (D) Images of a heterozygous wtf4antidote-GFP/ade6+ diploid cell and mature ascus. Wtf4antidote-GFP is shown in magenta. All Images were taken after 3 days on sporulation media. TL = transmitted light. All scale bars represent 2 μm. Images were taken with the same settings. Not all images are shown at the same brightness and contrast to avoid over saturation of pixels in the brighter images. (TIF) [file pgen.1009847.s002.tif]

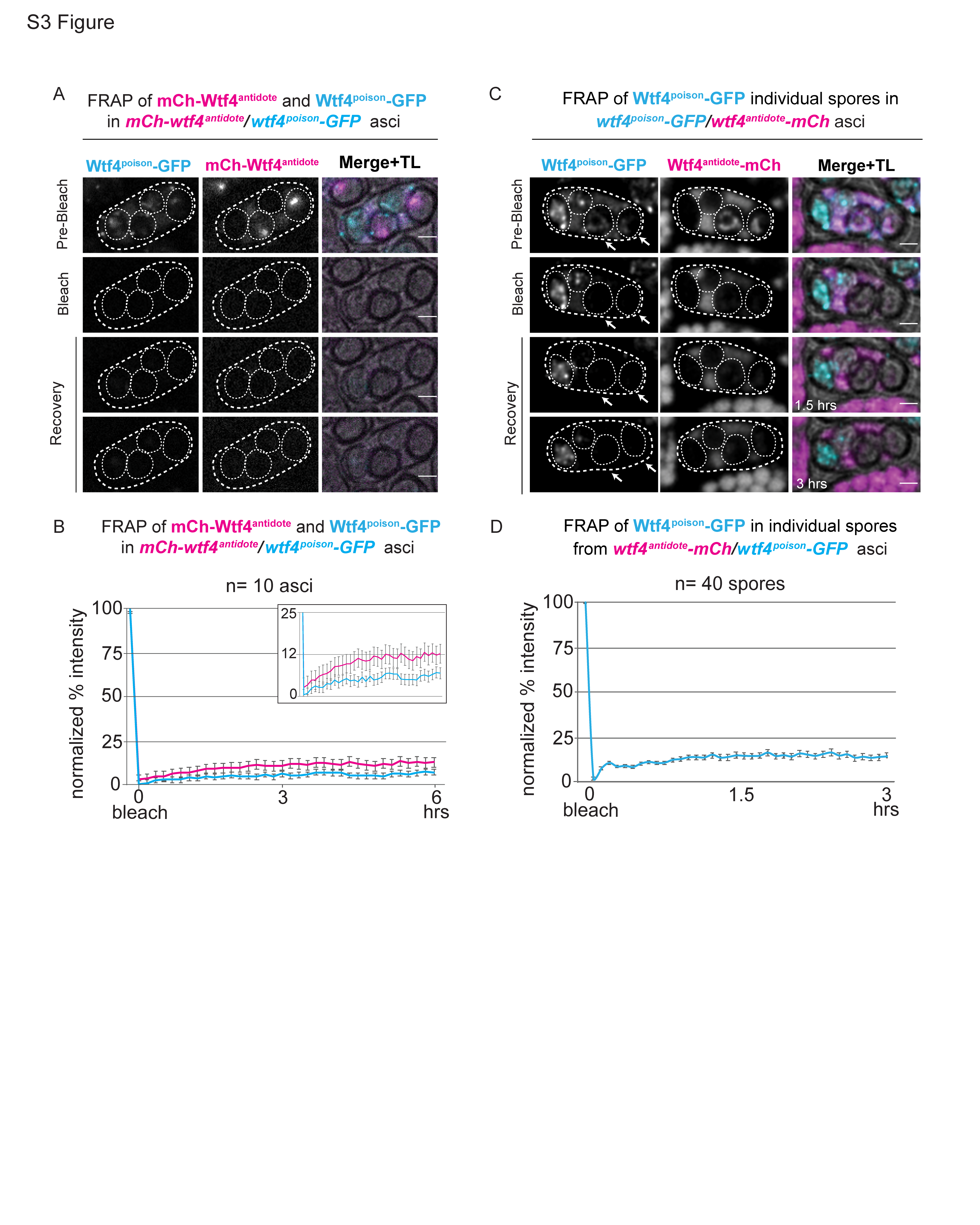

Supplement: S3 Fig — (A) Representative images from a Fluorescence Recovery After Photobleaching (FRAP) experiment with mature asci (n = 10) generated from mCherry-wtf4/ wtf4poison-GFP diploids. (B) FRAP of both mCherry (magenta line) and GFP (cyan line) post bleaching to 0% intensity and recovery quantified over 6 hours. (C) Representative images from a FRAP experiment with spores in mature asci (n = 40) generated from wtf4antidote-mCherry/wtf4poison-GFP diploids. (D) FRAP of GFP (cyan line) post bleaching to 0% intensity and recovery was quantified over 3 hours. Not all images are shown at the same brightness and contrast to avoid over saturation of pixels in the brighter images. (TIF) [file pgen.1009847.s003.tif]

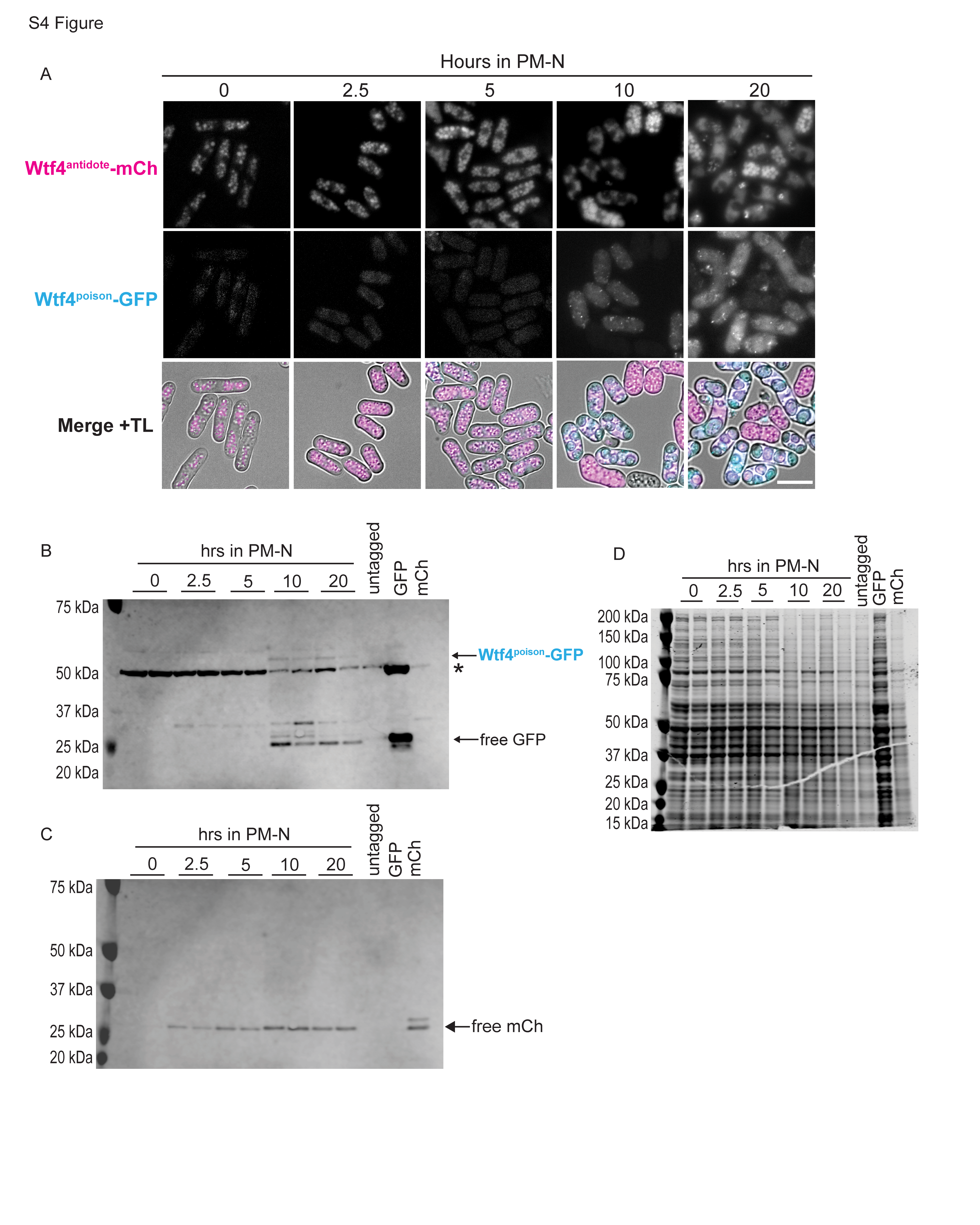

Supplement: S4 Fig — (A) Time course of wtf4antidote-mCherry/wtf4poison-GFP diploid showing the localization of mCherry-Wtf4antidote (magenta in merged images) and Wtf4poison-GFP (cyan in merged images) after the indicated times in PM-N media. All scale bars represent 10 μm. These are images of the cell populations sampled for the westerns. (B) Western blot of whole cell extracts from cells expressing wtf4poison-GFP at the times indicated. *Indicates a non-specific band. Two biological replicates are shown on the gel. (C) Western blot of whole cell extracts from cells expressing wtf4antidote-mCherry at the times indicated. (D) A replicate gel was stained with Imperial protein stain to show quantity of protein loaded. (TIF) [file pgen.1009847.s004.tif]

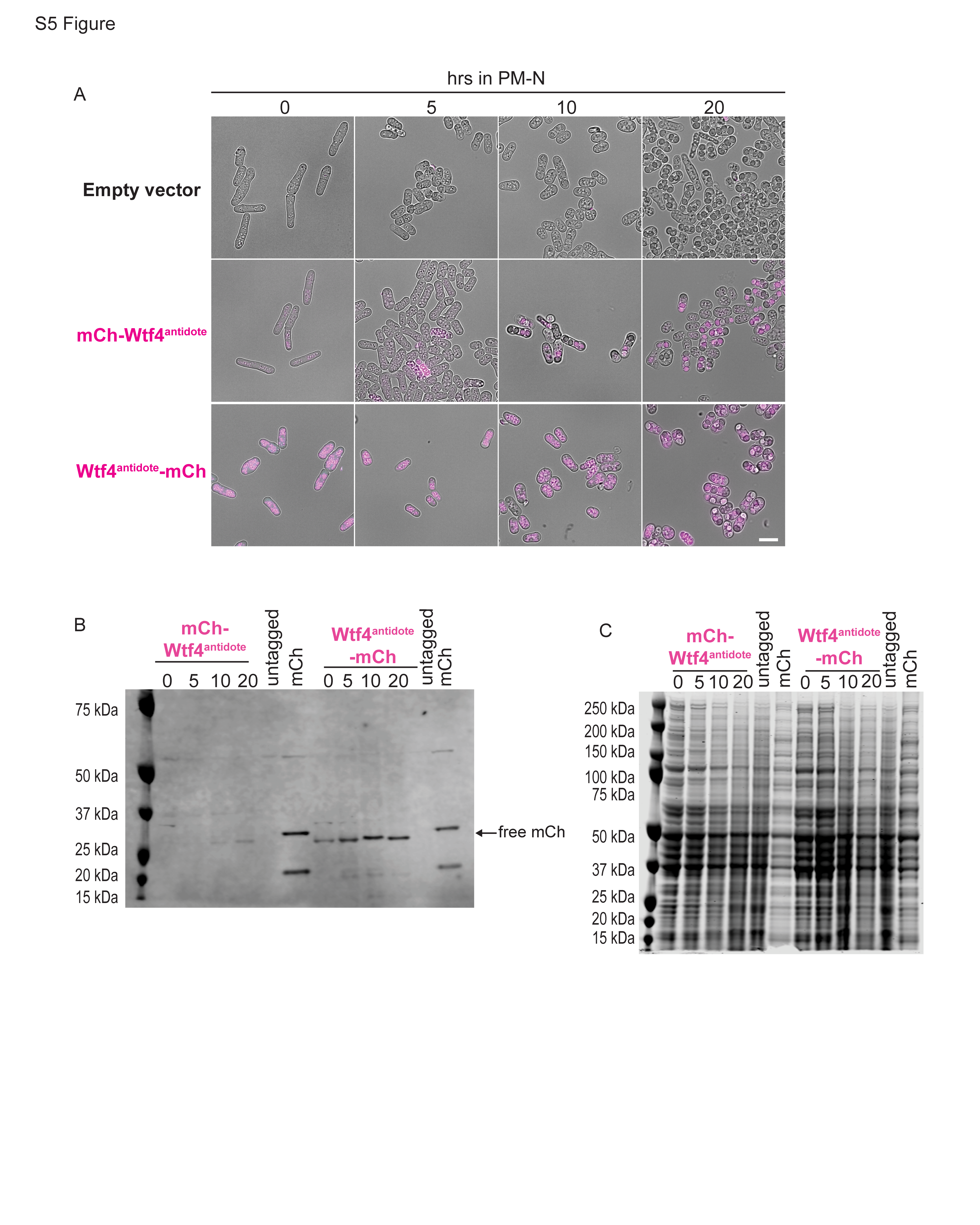

Supplement: S5 Fig — (A) Time course of mCherry- wtf4antidote/EV and wtf4antidote-mCherry/EV diploids showing the localization of Wtf4antidote (magenta in merged images) after the indicated times in PM-N media. All scale bars represent 10 μm. These are images of the cell populations sampled for the westerns. (B) Western blot of whole cell extracts from cells expressing either mCherry- wtf4antidote or wtf4antidote-mCherry at the times indicated. The mCh control was isolated from mitotically growing cells expressing constitutive mCherry. (C) A replicate gel was stained with Imperial protein stain to show quantity of protein loaded. (TIF) [file pgen.1009847.s005.tif]

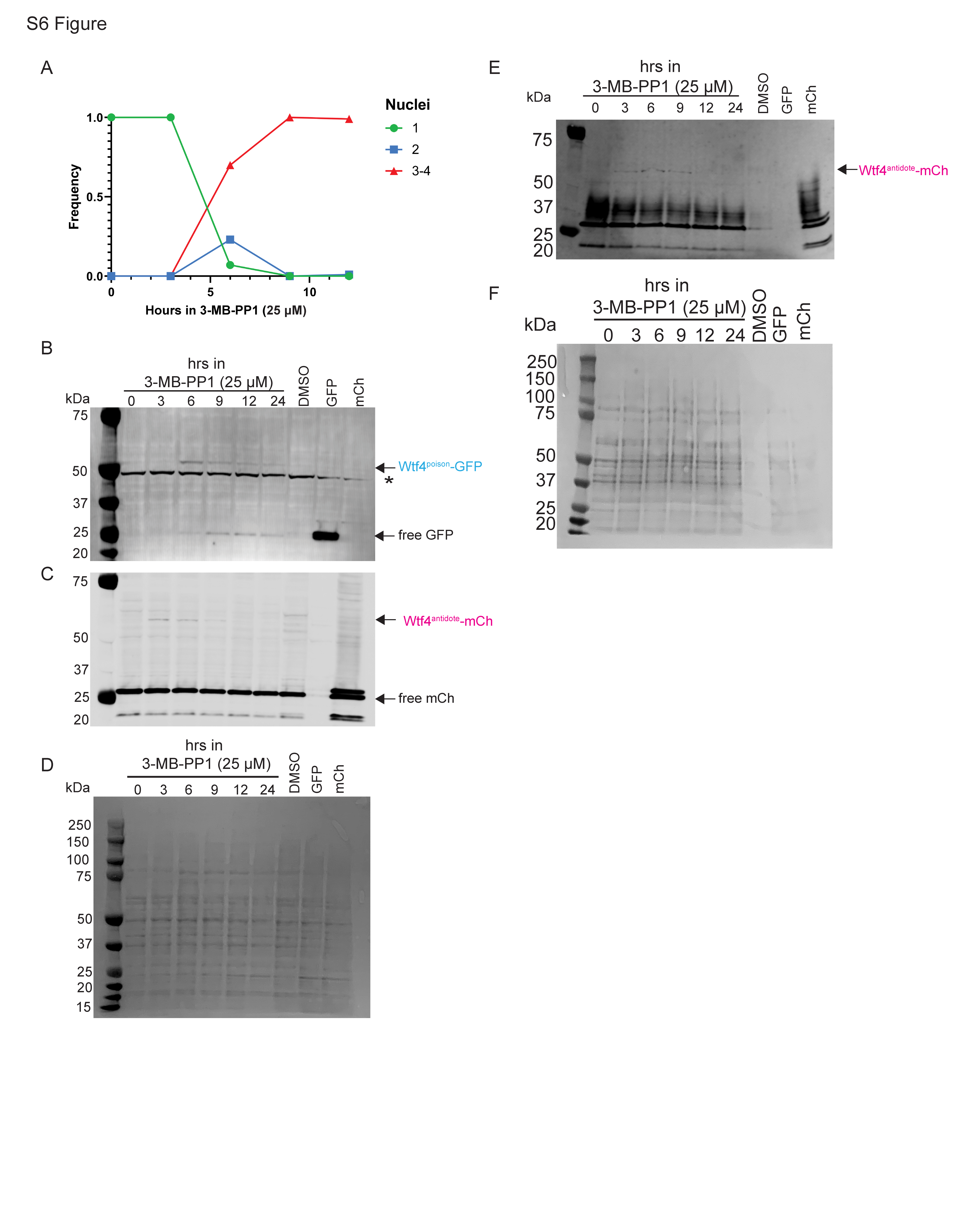

Supplement: S6 Fig — (A) Time course showing meiotic progression of h-/h- pat1.L95G/pat1.L95G diploid cells that were heterozygous for both wtf4poison-GFP and wtf4antidote-mCherry [57] 0–24 hours (top) following the addition of 3-MB-PP1 (25 μM) to induce meiosis. The number of nuclei in DAPI-stained cells was counted at the indicated time points. (B) Anti-GFP Western blot of whole cell extracts of cells from the same experiment depicted in A. Bands consistent with Wtf4poison-GFP and free GFP are highlighted with arrows. *indicates a non-specific band. The GFP and mCherry controls were prepared from sporulated cells expressing the free fluorophores constitutively (SZY2636 and SZY2638; see methods). The DMSO control was prepared from a diploid with the same genotype as the experimental sample, but treated for 24 hours with DMSO instead off 3-MB-PP1. (C) Anti-mCherry Western blot on the same membrane as that shown in B. Bands consistent with Wtf4antidote-mCherry and free mCherry are highlighted with arrows. (D) Ponceau staining of the membrane in B-C. (E) The same protein samples used in the blot in B-C were rerun on a new anti-mCherry Western where blotting stringency was increased by the addition of 0.2% Tween 20 to the primary antibody. A band consistent with Wtf4antidote-mCherry is observed and is not present in the negative control lanes. (F) Ponceau staining of the membrane shown in E. The DMSO control sample was erroneously not loaded on this blot. (TIF) [file pgen.1009847.s006.tif]

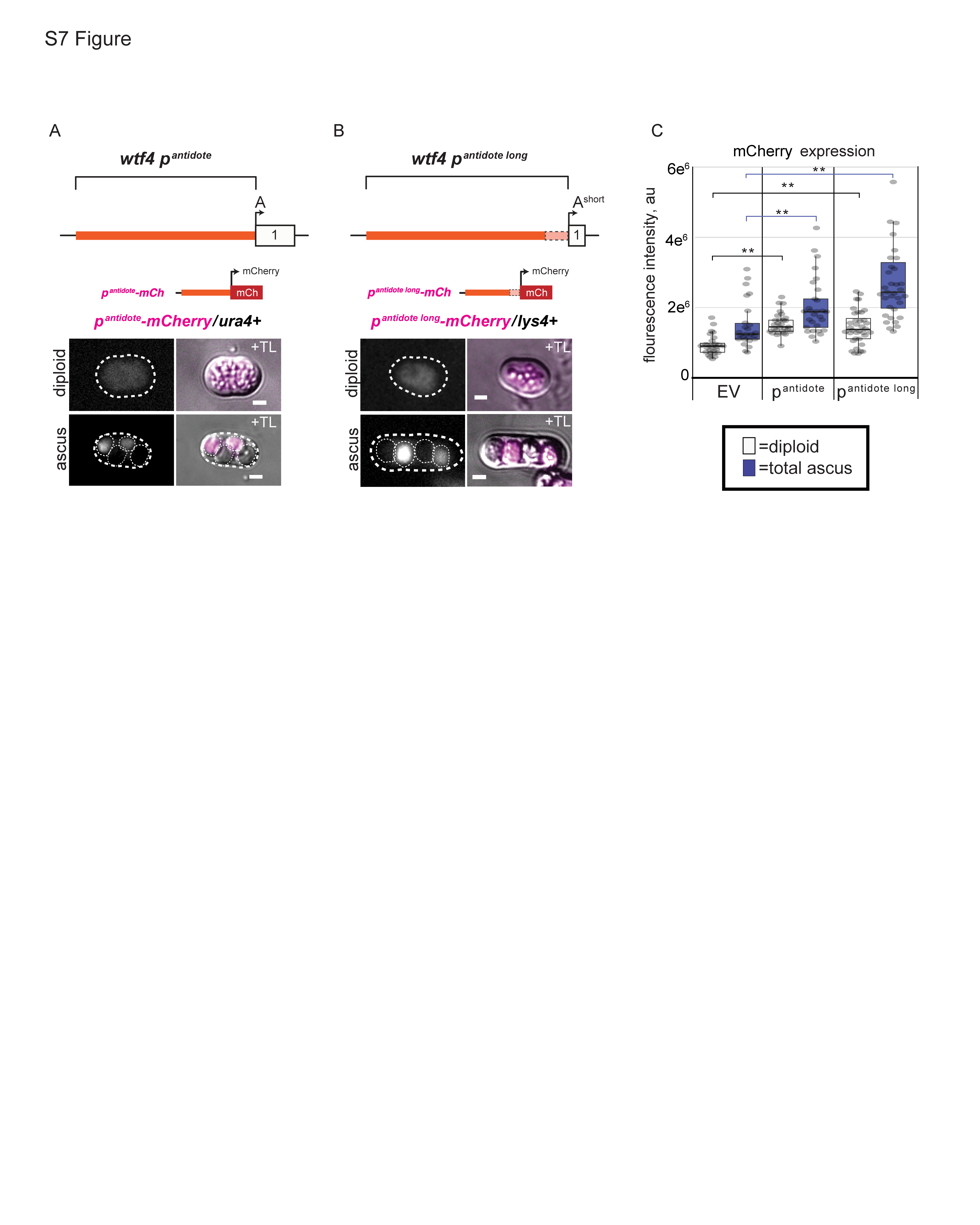

Supplement: S7 Fig — Depictions and images of the two lengths of wtf4antidote promoters used in this study, pantidote (A) and pantidote long (B). The images of the two different pantidote-mCherry reporters / + were from heterozygous diploids and asci. (C) Quantification of mCherry fluorescence within heterozygous (pantidote-mCherry reporter/ +) diploids and asci. At least 25 diploids and 25 asci were quantified per reporter. All images were acquired after 3 days on sporulation media. TL = transmitted light. All scale bars represent 2 μm. Images were taken at the same settings and are shown at the same brightness and contrast for accurate comparison. (TIF) [file pgen.1009847.s007.tif]

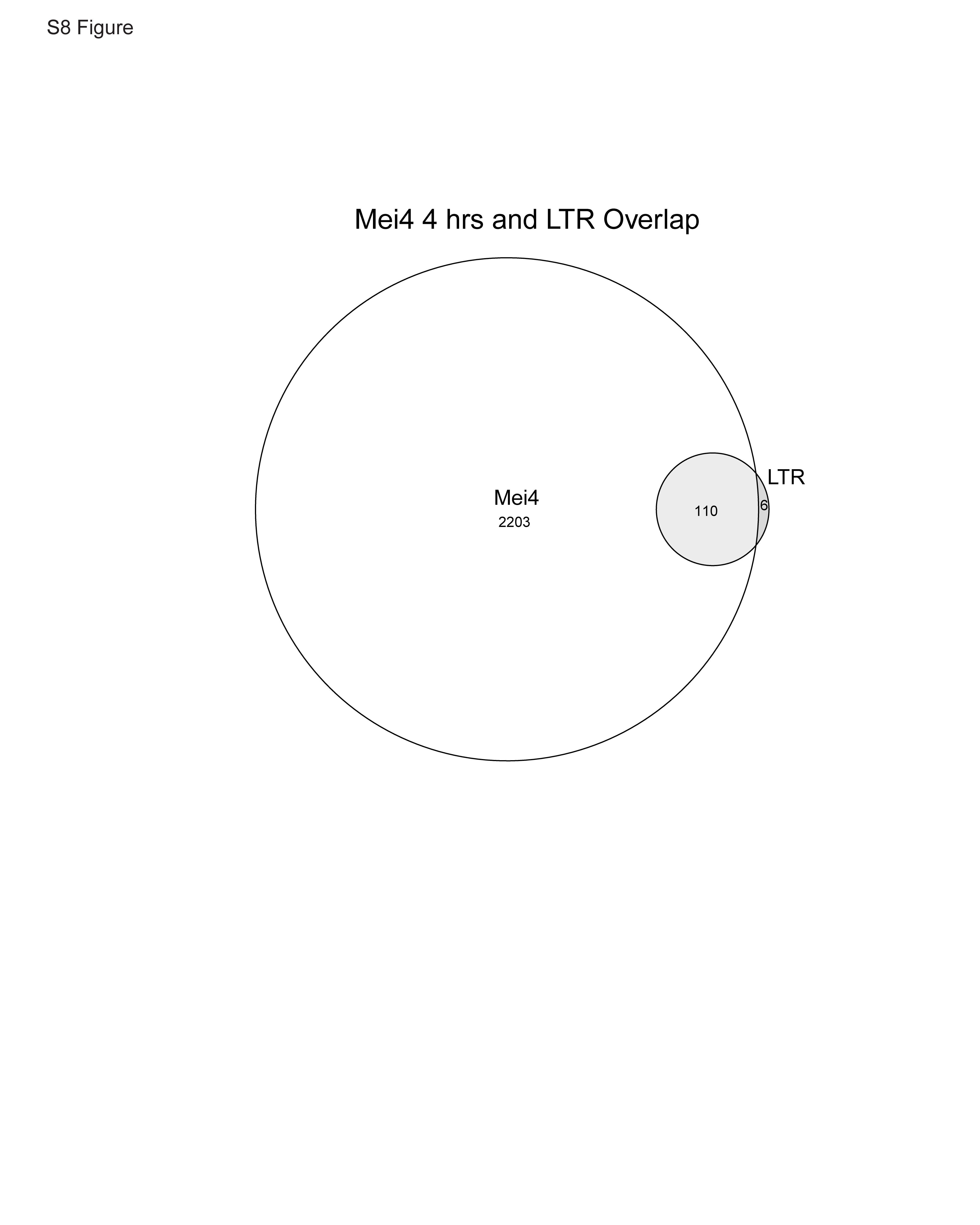

Supplement: S8 Fig — Peaks (called by MACS2) from Mei4 ChIP-seq (data from [50]) 4 hours past meiotic induction are compared to LTR regions (identified by BLAST). If the regions overlap, they are shown in the shared region of the Venn diagram. For any ChIP-seq reads that mapped to more than one location, only a single location, chosen at random, was selected. (TIF) [file pgen.1009847.s008.tif]

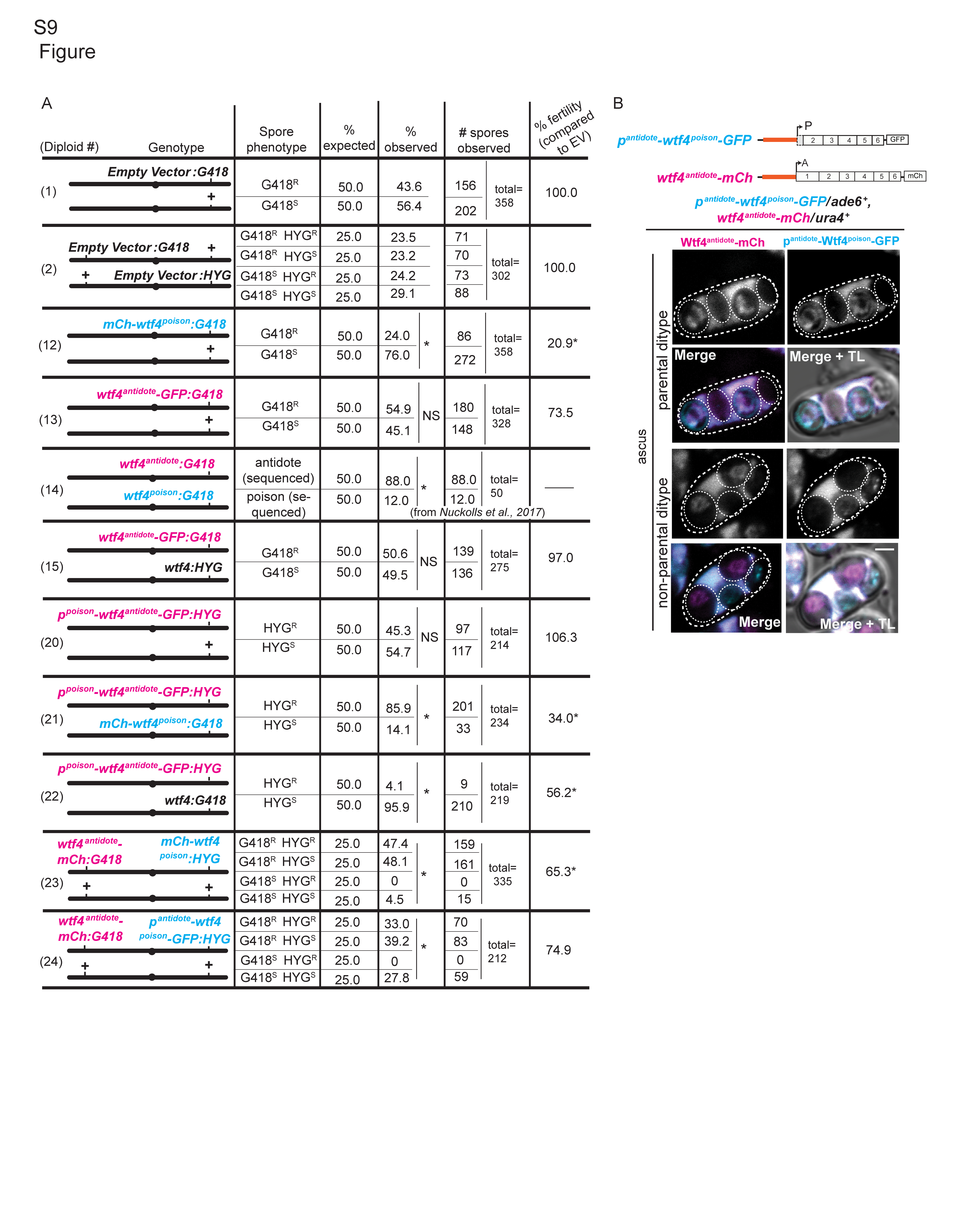

Supplement: S9 Fig — (A) Allele transmission and fertility (assayed via viable spore yield) of 11 diploids of the depicted genotypes. The genotype column shows a cartoon depiction of the relevant genotype. The progeny phenotypes are then shown on the right. For diploids heterozygous at one locus (e.g., diploid 1), two values are shown (top and bottom) that represent the two possible haploid genotypes. For diploids heterozygous at two loci, the loci used are unlinked and should segregate randomly. The depictions are not to scale with the location of the loci on the chromosomes. Spores exhibiting both parental phenotypes were considered diploid or aneuploid and were excluded from this table but can be found in S1 Data. The expected values assume Mendelian allele transmission. (* = p < 0.05, NS = not significant; G-test for allele transmission, Wilcoxon test for VSY, in comparison to the empty vector control). We compared diploids 12, 13, 14, 20 to diploid 1 as the control and diploids 15, 21, 22, 23, 24 to diploid 2 as the control. The data for the control diploids 1–2 and 12–15 are also depicted in S2A Fig. The data for diploid 1 is also in Fig 3C. (B) Images of pantidote-wtf4poison-GFP/ade6+, wtf4antidote-mCherry/ura4+ parental and non-parental ditype asci. pantidote-Wtf4poison-GFP is shown in cyan and Wtf4antidote-mCherry is shown in magenta in merged images. TL = transmitted light. All scale bars represent 2 μm. All images acquired after 2 days on sporulation media. All images are shown at the same brightness and contrast for accurate comparison. (TIF) [file pgen.1009847.s009.tif]
