# Supplementary material for: S. pombe wtf drivers use dual transcriptional regulation and selective protein exclusion from spores to cause meiotic drive
Source: PLoS Genet. 2022 Dec 7;18(12):e1009847. doi: 10.1371/journal.pgen.1009847 (PMC9762604; doi:10.1371/journal.pgen.1009847)
Supplement: Attachment — Submitted filename: Response to reviewers.docx [file pgen.1009847.s016.docx]

Dear Greg,

We wish to thank you and the reviewers for your time in evaluating our submission and for your patience during extended time it took for us to complete additional experiments and extensively revise the manuscript. We appreciate the thoughtful feedback and we used it to improve the paper. These were truly helpful reviews! We have responded to each of the reviewer comments and queries below in blue indented text. Please note that the figure quality is poor in the system generated PDF and we could not resolve this problem. The high-res versions are available to the reviewers in the file inventory.

SaraH

Reviewer's Responses to Questions

Comments to the Authors:

Please note here if the review is uploaded as an attachment.

Reviewer #1: In fission yeast, wtf genes are meiotic drivers that kill the spores that do not inherit the genes from a heterozygote diploid. Here, the authors focus on wtf4, showing that wtf4-poison and wtf4-antidote are products from a single gene (with wtf4-poison ORF totally included in wtf4-antidote), but with different transcriptional profile. This fact ensures that only spores that have wtf4 gene and express wtf4-antidote (once the spores are formed) will be able to survive.

My major concern with this manuscript is that I do not find any substantial advance over previous manuscripts from the same group (Nuckolls et al, eLIFE, 2017 & 2020); however, there is plenty of room for improvement given the observation that delta-FLEX has no expression of wtf4-poison in meiosis.

This paper employs several alleles used in previous analyses. In the new draft, we made more effort to clarify what we knew prior to the experiments presented in this manuscript. The experiments presented in this paper support the following conclusions that are new to this work and important when considering the mechanism and evolution of *wtf* drivers:

1) That two distinct promoters combined with distinct behaviors of the two Wtf4 proteins at the time of spore formation are responsible for the distinct localization patterns of the proteins observed in asci.

2) That the two Wtf4 proteins are effective under the control of their own promoters, but do not function well under the control of the promoter that controls expression of the alternate protein.

3) That Mei4 controls expression of the Wtf4 poison protein.

Could the authors introduce a FLEX element upstream of wtf4-antidote and neutralize wtf4-poison?

This experiment mentioned was present in the original submission, but was poorly presented. Because of that, we extensively rewrote the paper. Regarding this specific comment, our experiments used an allele that expressed the *wtf4^antidote^* from the poison promoter (with FLEX element). This allele did not neutralize the poison based on the genetic analyses presented. We saw with this allele and others that the Wtf4^antidote^ expressed prior to spore formation is largely excluded from spores, whereas the Wtf4^poison^ present at the same time is less likely to be excluded. This likely explains why spore-specific expression of the antidote is necessary.

In the revision, we have attempted to communicate the experiments and results more clearly. In most figures, we have added cartoons to illustrate the nature of all the alleles tested. In addition, we added a new figure (S1) that summarizes all the alleles tested.

Can delay wtf4-poison by replacing FLEX element with an element to induce the gene later in meiosis?

This experiment was also present, but not clearly explained in the last draft. We see a burst of expression from the *p^antidote^* promoter shortly after spore individualization (e.g. video S1 and figure 2) that is not observed with the *p^poison^* promoter. When we express the Wtf4^poison^ protein from this promoter, the Wtf4^poison^ was not effective at poisoning spores beyond those spores in which the protein was expressed (S7 fig diploid 24). This made the allele defective in causing meiotic drive.

Another important point is that all tagged strains should be quantitatively validated on WB on synchronous meiosis (for example, in a pat1-ts strain), determining poison and antidote. There is not a single WB in the whole manuscript

***pat1*-ts:** We chose to use wild-type cells for our analyses, rather than rather than *pat1* mutants, to image cells under the same genetic and experimental conditions used to assay meiotic drive in this and other studies. In addition, our claims focus on pre- and post-spore individualization. Because of this, the visual landmarks of spore formation worked well to stage the cells for our analyses (e.g. before and after spore formation).

**Validation of tagged strains:** Our claims focus on function, rather than protein abundance. It is possible that the tags affect protein abundance, but it was not to such an extent that function of the alleles was greatly compromised when they were expressed from their endogenous promoters. We validated all of our tagged strains functionally using genetic analyses (S2 Fig, Fig3, S7 Fig). We also showed that our claims regarding differential exclusion of Wtf4^poison^ and Wtf4^antidote^ proteins from spores is supported by multiple tagged (GFP and mCherry) alleles for both proteins (e.g. S2 Fig C-D, Fig 4-5). When we swapped promoters expressing the Wtf4 proteins, we compared those alleles functionally to controls with endogenous promoters (S7 Fig).

**Westerns:** We now include western blots in the paper. Not, however, to the extent suggested by the reviewer both because we prefer functional validation of alleles and because Wtf proteins are challenging to interrogate via western blotting. First, the proteins are incredibly hydrophobic aggregating proteins that may also be membrane-associated (Wtf4 has 7 predicted transmembrane domains). Such proteins can be difficult to assay via western blotting (DOI: 10.1016/j.ab.2008.10.005). After extensive trials, we can detect a band consistent with Wtf4^poison^-GFP. Another reason westerns are challenging for Wtf4 proteins is due to their mechanisms. The Wtf4^antidote^ is quickly trafficked to vacuoles, where it presumably has limited stability. When the Wtf4^antidote^ is present, Wtf4^poison^ is also quickly taken to the vacuole. Likely because of this, we largely detect free tag on western blots rather than Wtf4protein+tag.

This free tag on a western blot can reasonably lead one to suspect that the tags we observe in cells are free fluorophore and are not associated with Wtf4 protein. We know this is not the case for Wtf4 proteins from our prior work (Nuckolls et al. 2020). There are many experiments supporting this presented in that paper, but I will mention a

few to put the reviewers at ease. 1) Free fluorophores are soluble, but tagged Wtf4 proteins assemble into aggregates. [You can also see the difference by comparing Figure 1C and Fig 2 in this work.] 2) We can cause a re-localization of the tagged Wtf4^poison^ aggregates to the vacuole by expressing tagged or untagged Wtf4^antidote^ protein. 3) Tagged Wtf4 proteins colocalize and show FRET in cells. Free fluorophores do not FRET and are not expected to re-localize upon Wtf protein expression.

Minor points

Figure 2B and 2D: the figures should extend the sequence, to include contigous sequences.

We extended the sequence region shown as suggested.

pg. 10, first paragraph; the authors reference to Nuckolls 2017 to mention that a fully functional wtf4-antidote can be made from a second ATG… However, I could not find the experimental validation for that sentence

Thank you for pointing this out. We revisited the paper and realized we mentioned this in Nuckolls 2017, but we did not provide the data to support the statement in that paper. We have now presented it in S2 fig.

pg. 13, first paragraph: the authors wrongly call figures 4D and 4E

Thank you, the figures have been extensively changed and this typo is now gone.

pg. 16-17: if the authors want to quantitate the expression of the different promoters, they should do Q-PCR, not follow the expression by Cherry fluorescence.

We chose the transcriptional reporter over Q-PCR to easily enable higher resolution consideration of single cells, rather than averaging a bulk population. This helped us stage cells without using *pat1* mutant meiosis.

Reviewer #2: In their article on S. pombe wtf meiotic drivers, Nuckolls et al. investigate the wtf4 spore killer. Previous results have shown that the wtf4 spore killer makes a poison protein and an antidote protein from two distinct but overlapping transcripts. With fluorescent microscopy, Nuckolls et al. provide evidence that the Wtf4 poison is distributed evenly in all four spores of a wtf4/+ heterozygote, while the Wtf4 antidote in enriched in two of the four spores. The authors also show that expression of the Wtf4 antidote can be induced by stress. Finally, they show that expression of the Wtf4 poison is likely controlled by Mei4 (a critical meiotic-stage transcription factor). I found the article to be interesting and the results to be valuable with respect to understanding wtf4-based meiotic drive.

I have some concerns that the authors might want to address in a revised manuscript.

My first concern is with the authors interpretation of the conflicting results with respect to the N- and C-terminal mCherry tags of the Wtf4 antidote. In lines 196-199, the authors write “We conclude that the C-terminal tag reveals the production of a Wtf4 protein population that is not apparent with the N-terminally tagged allele. Although the nature of the additional protein is not clear, it could be produced using the second translational start site found in exon 1 (codon 12)”. I find this explanation to be confusing. Are the authors proposing that the beginning of antidote exon 1 serves as an internal ribosome entry site? Can they clarify their explanation and cite a similar example where a fluorescent protein coding sequence was bypassed in a transgene? If not, perhaps it would be useful to examine the result of fusing mCherry to the second translational start site.

Thank you for pointing this out. We have tried to make this more clear in the revision. When we use C-terminally tagged alleles, we see earlier Wtf4^antidote^ signal than with N-terminally tagged alleles (Fig 1). It is possible the early protein visible with the C-terminally tagged allele could be produced from the second ATG in the coding sequence. This could occur via promiscuous choice of start codon (e.g. DOI: 10.1093/nar/gkl280). Such a protein would not be visible with our N-terminal tag. It is also possible that the early population of Wtf4^antidote^ is produced from the first start codon, but that its production is disrupted by the N-terminal tag. We did not distinguish between these possibilities.

My second concern is that the authors do not consider the roles of 5’ UTRs while interpreting their results. For example, the antidote promoter (i.e., the 285 bp of sequence found immediately upstream of the antidote start codon) should produce an mRNA with an antidote 5’UTR while the poison promoter (i.e., the 230 bp of sequence found immediately upstream of the poison start codon) should produce an mRNA with a poison 5’ UTR. These UTRs should also be present in the mCherry and GFP mRNAs expressed by the Pantidote-mCherry and Ppoison_GFP constructs. Could the antidote and poison 5’ UTRs be major controllers of when poison and antidote are expressed during ascus development and where the proteins are found within asci? If so, can the authors shed light on how this could influence the interpretation of their results?

This is a good point and a limitation of our study. We did not experimentally separate the roles of those sequences as promoters from their roles as UTRs. We now mention these limitations in the paper.

Regarding localization of the proteins, we added additional photobleaching experiments and clarified the writing. Our results show that most of the Wtf4^poison^ protein is made prior to spore formation and doesn’t exchange between spores or between spores and ascal cytoplasm (Fig 1, S3 Fig). For the Wtf4^antidote^ protein, we show the protein present prior to spore formation tends to be excluded from spores. The simplest model explaining enrichment of the Wtf4^antidote^ protein in the two spores that inherit the locus is transcription of the locus in those spores.

My third concern deals with transport of proteins between delimited spores of an ascus. My interpretation of the model as presented in the manuscript is that all poison inside non-wtf4 spores must get into these spores before they are delimited. But this is based on the assumption that the poison cannot be transferred from wtf4 spores to non-wtf4 spores after spore delimitation. Are the authors sure that the poison cannot be exported from wtf4 spores after spore delimitation?

Thank you for pointing this out. We tested this experimentally via photobleaching (S3 Fig) and we do not see transfer of either Wtf4 protein into, or out of, spores.

My final concern is with the Mei4 findings. While I agree with the authors that Mei4 likely controls poison expression, the claim in lines 35 to 36 is too strong. For example, the authors state that “we show that the Mei4 transcription factor, a master regulator of meiosis, controls the expression of the wtf4 poison transcript”. Currently, this claim is mostly based on the inability of wtf4 to drive when a putative Mei4 binding site in the poison promoter is deleted. I think this a claim of this magnitude should be supported with biochemical experiments. Alternately, the authors could simply improve the manuscript by changing the text to something like “our results strongly suggest that Mei4 controls…”.

Our claim is based on our data showing that an allele lacking the FLEX motif acts as an antidote-only allele (protects against drive, but doesn’t drive). This shows it makes a functional Wtf4^antidote^, but not a Wtf4^poison^. We also provide biochemical support for this claim in our analysis of a pre-existing ChIP-seq data set that shows Mei4 binds with intron 1 of *wtf* genes that encode poison proteins (i.e. poison promoters), but is not associated with intron 1 of *wtf* genes that do not encode poisons. Finally, our conclusion is also supported by additional genetic data from others cited in the paper in which *wtf* gene expression was decreased when *mei4* was deleted and increased when *mei4* was over-expressed.

Other comments:

Line 120 – please check formatting of loannoni citation

We have corrected formatting problems in the references.

Line 126 – please confirm text, it currently suggests that S. kambucha is a subspecies of S. pombe, is this true?

Species concepts are contentious. We refer to *S. kambucha* as natural isolate of the *S. pombe* species. It has its own name because it was first designated as a distinct species because hybrids generated by crosses between *S. kambucha* and the lab isolate of *S. pombe* are infertile because of rampant meiotic drive (biological species definition). But under the biological species definition, most *S. pombe* isolates could be considered distinct species, even though they have low diversity (outside of rapidly evolving regions, like *wtf* drivers) because hybrids are infertile.

Line 131, Figure 1A, etc. I think that it is standard practice to consider anything that remains in a transcript after intron removal to be part of an exon.

We agree with this practice, but there is a wrinkle in applying it to *wtf4.* Some sequence that is part of intron 1 of the *wtf4^antidote^* transcript is UTR and codon in the *wtf4^poison^* transcript. We have illustrated the sequence that is alternately intron and codon in the dotted box in our figures and added additional illustrations to the paper to clarify the nature of the alleles tested.

Line 473 – please check that the statement is accurate. For example, why “must” all spores be exposed to a lethal dose, couldn’t only the killed spores be exposed to a lethal dose?

Good point ‘must’ is not the correct word. We have changed this section to be more precise.

Line 1114 – please check bold font in ref 94.

We have corrected formatting problems in the references.

Reviewer #3: This group has enjoyed a series of high quality and informative papers on this very interesting topic over the last several years. This manuscript unfortunately does not represent a continuation of that trend. There is a lot of redundancy with previous papers, to the point of showing replicate data from a previously published paper (Fig 1C, which to the authors credit, they point out) More importantly, this manuscript suffers from some lower quality data which poises some problems for the interpretations.

Some of the genetic data were presented in previous work. These experiments serve as controls or important reference points for the additional work presented in this paper so we present them, with references here. In addition, we did previously assay the mCh-*wtf4/wtf^poison^-GFP* diploid. We clarify in the revision that our previous analysis of this diploid was limited. We now expand that analysis by following the signal from the two tagged Wtf4 proteins through a time-lapse (bulk population; Figure 1C, and individual cells, video 1). We agree with the reviewer that his analysis, alone provides minimal new information. However, we felt it was important to present these data because the analyses of new tagged antidote alleles presented in this work (Fig 1E and S2 Fig) show that the previously used mCh-wtf4 allele fails to reveal the entire scope of Wtf4^antidote^ expression and localization. We felt readers would need to compare the tagged alleles side-by-side to appreciate the differences.

The reviewer is correct that some of the images in the original submission were low resolution. We have repeated these experiments again and taken higher resolution images. The conclusions remain the same, but the data are now easier to interpret.

The bigger problem is conceptual. There is no good reason to presume that wtf-poison needs to be opposed during early meiosis as the authors put forth as a guiding premise. The period of time in which poison is unopposed during meiosis is transient, and the fact that wtf-poison expression is toxic when chronically expressed in mitotic cells does not provide a sound rationale for the presumption of wtf-antidote function during early meiosis.

We have changed the narrative arc of the paper and focused our claims.

The authors are able to engineer an antidote protein allele leading to its accumulation in the cytosol of meiotic cells which bolstered their premise. The problem is that the data uphold the conclusion that the intronic sequences separating exons 1 and 2 contains information that represses transcription upstream of exon 1. This could be simply explained by a transcriptional interference mechanisms whereby Mei4 blocks elongation of the longer transcript, though there are numerous other possibilities.

We disagree with that the data supports a transcriptional interference model, or that such a model explains our observations. We see spore-specific mCherry signal in both N-terminally tagged *wtf4^antidote^* alleles that do and do not have intron1, suggesting the intron (or Mei4 binding) is not responsible for this pattern (Fig 1C and S2 Fig B). Similarly, we see Wtf4^antidote^ signal prior to spore formation from C-terminally tagged Wtf4^antidote^ alleles that do, and do not, contain intron 1 (Fig 1E and S2 Fig C-D). These observations, in addition to previous long-read RNA sequencing data referenced in the paper support our claim that there is expression of Wtf4^antidote^ prior to spore formation. The toxicity of the *p^antidote^-wtf4^poison^* allele described in this work also supports our conclusion that Wtf4^antidote^ is not only expressed in spores.

It is possible that the level of *wtf4^antidote^* transcription varies in the presence/absence of intron 1 as suggested by the reviewer. Given that we see no phenotypic differences in meiotic drive between *wtf4^antidote^* alleles that do contain an intact intron 1 compared to those that do not, we did not pursue these potential differences

Accordingly, the image quality of the pictures of meiotic cells expressing the wtf-antidote-mCherry allele are poor. It is pointed out in the Fig 1 legend that imaging conditions are not uniform throughout, making it very challenging for the reader to make an apples to apples comparison in order to assess the conclusions. One additional way through this would be to provide some other means of quantification of relative abundances, such as western blotting. Whatever the case may be, the relative amounts of antidote accumulating in the cytosol during early meiosis and in the spores following the completion of meiosis are dramatically different when comparing the mCherry-wtf4 vs wtf4-mCherry-antidote. Independent of the "apples to apples" comparison problem of mCherry-wtf4 vs wtf4-antidote-mCherry, this internal comparison of relative accumulation within each experiment informative and poses a challenge to much of the manuscript: relative to antidote accumulation in the developed spores, wtf-antidote-mCherry produces dramatic accumulation of wtf4-antidote in early meiotic cells in marked contrast to that observed using the more native mCherry-wtf4 allele. This is not adequately explained, and as described above, it seems like the best explanation for this is the that the intronic sequences removed from the wtf4-antidote-mcherry allele lead to de-repression of transcription of the longer antidote-encoding transcript. Thus, the premise for the rest of the paper is dubious.

We have repeated the experiment mentioned and carried out time-lapse image analyses. We use this experiment to provide additional quantification of mCherry and GFP in figure 1. These time courses were carried out in parallel and imaged under the same conditions. Both experiments also include the *wtf4^poison^-GFP* allele, giving a relative anchor point to help one compare the expression timing of the two mCherry alleles.

See comments above regarding western blotting.

This is not adequately explained, and as described above, it seems like the best explanation for this is the that the intronic sequences removed from the wtf4-antidote-mcherry allele lead to de-repression of transcription of the longer antidote-encoding transcript. Thus, the premise for the rest of the paper is dubious.

The narrative arc of the paper has been changed. It now longer focuses on why diploids do not die as they transverse meiosis full of poison as the driving premise. Now, we demonstrate the expression of both poison and antidote proteins occurs before and after meiosis, we show that the differential control of the two proteins is functionally important, demonstrate the two proteins behave differently during spore packaging, and we show that Mei4 controls the expression of the poison protein.

Additional comments.

This work would really benefit from the use of Northern blots to display the two transcripts and their kinetics during meiosis. I realize the existence of the 2 transcripts is now well supported from analysis of genomic data sets and additional experiments. However, given the centrality of the role of the two wtf4 transcript isoforms, and the use now of so many constructs deleting and/or adding information to these transcripts, it is my view that Northern blotting is an essential complement to the authors approaches. For example, does deletion of intron 1 (or just the Mei4 target sequence) lead to de-repressed transcription from the longer isoform?

We see no phenotypic difference in meiotic drive between *wtf4^antidote^* alleles that do contain intron 1 compared to those that do not. Thus, any potential differences in transcript levels between the different antidote alleles driven by their endogenous promoter do not significantly affect the process we are studying, meiotic drive. Because of this, we did not explore this possibility.

The alleles that do modify the meiotic drive phenotypes are those in which we have swapped the promoters driving the expression of the Wtf4^poison^ and Wtf4^antidote^ proteins. We claim that protein localization is altered with these alleles and that their function in drive is compromised. These conclusions are supported by the data we present.

Northern blots in synchronous meiosis would help characterize the precise differences between the two transcripts, which is valuable information, but not essential for the claims we make. In addition, completing this analysis would require us to do substantial additional work. We would need to reconstruct many alleles in a *pat1* mutant background and test if drive is a phenotype disrupted by *pat1* mutation, in addition to the northern blot experiments. Finally, the published data regarding the two *wtf* transcripts cited in the paper is quite rich (DOI: 10.1101/gr.208041.116). It is long read RNA sequencing data taken from a meiotic time course. The long reads help distinguish between the long and short transcripts, and between the 25 different *wtf* genes.

For 2G. Is the poison-promoter-GFP seen in spores really representative of “expression” within them, or just inheritance of GFP from reporter expression during meiosis?

It is consistent with inheritance of the GFP signal from the diploid as we assayed diploids heterozygous for both reporter constructs. With the antidote promoter, we observe 2:2 spore enrichment. With the poison promoter, we see more even distribution between the spores.

3A already shown in 1D. exact same result.

This was intentional and meant to make the paper easier to read by enabling side by side comparisons of the relevant images. We maintained this redundancy between figure 1 and what is now figure 5. We used different images for the two figures to illustrate the consistency of the patterns. We also maintained redundancy in our genetic tables to hopefully enable easier reading.

3B. wtf-antidote under a wtf-poison promoter. exon 1 sequences (RNA or its encoded protein), prevent poison from being imported into spores. Not explained well by authors.

Thank you for pointing this out. We have extensively rewritten this to make it more clear.

Authors argue that promoter-reporter constructs recapiluate 1D, but this is not really the case. The wtf4-antidote-mcherry reporter shows much more convincing expression in meiotic cells than the antidote-promoter-GFP reporter.

We agree with the reviewer and have modified the text.

Why does diploid 7 show a reduced frequency of G418/Hyg sensitive spores?

In the new submission, diploid 7 is now diploid 9. The class of spores that is underrepresented is the G418^S^ HYG^S^ class. The *wtf4:HYG* allele encodes a poison and an antidote. The *wtf4^antidote^-mCherry:G418* allele encodes only an antidote. The underrepresented spores are those that do not inherit an antidote allele, and thus they succumb to the poison. We now include an additional figure (S1) to help clarify the proteins encoded by each allele.
