# Supplementary material for: S. pombe wtf drivers use dual transcriptional regulation and selective protein exclusion from spores to cause meiotic drive
Source: PLoS Genet. 2022 Dec 7;18(12):e1009847. doi: 10.1371/journal.pgen.1009847 (PMC9762604; doi:10.1371/journal.pgen.1009847)
Supplement: Attachment — Submitted filename: Response to ReviewsRH.docx [file pgen.1009847.s017.docx]

Dear Greg,

Thank you for the opportunity and time required to revise our paper. We have improved the draft since the last submission and have responded to each comment in indented blue font. I would also like to note that this submission corrects an error we missed in previous submissions in that the *wtf4^poison^* allele in diploid 23 (Figure S10) is tagged with mCherry, not GFP. The affected experiment (described on page 17 of the revision) is a genetic cross (not imaging) and both types of tagged *wtf4^poison^* alleles are functional, so it does not alter the conclusions of the experiment. We appreciate the time and efforts of you and the reviewers in helping improve our work.

Editor comments:

1. Attempting western blot quantification with highly synchronous background (like pat1). I ask that you consider trying to address this concern. Like the reviewer, I understand that using a different background from the other experiments is sub-optimal, but readers could be alerted to that caveat in the text.

We built new diploid strains containing the analog sensitive *pat1.L95G* allele in addition to our *wtf4^poison^-GFP* and *wtf4^antidote^-mCherry* alleles. We then induced this strain to undergo meiosis synchronously using an ATP analog. Our anti-GFP Western to observe Wtf4^poison^ (new S7 Figure) was not markedly different from the anti-GFP Western in *pat1+* cells (S6 Figure). Specifically, both types of experiments show induction of GFP signal in meiosis, with more free GFP than full-length Wtf4^poison^-GFP.

Our anti-mCherry Westerns to visualize Wtf4^antidote^ in *pat1.L95G* (S7 Figure) are also consistent with our previous results in *pat1+* cells. Specifically, we observe mCherry signal throughout meiosis. In *pat1+* cells, we only observed free mCherry (S5 and S6 Figures). In *pat1.L95G* cells, most of the signal is from free mCherry, but there is a faint band that could be full-length Wtf4^antidote^-mCherry.

These new observations are consistent with our imaging data, our claims regarding the expression of Wtf4^antidote^ prior to spore formation, and our previous observations that co-expressed Wtf4^antidote^ and Wtf4^poison^ proteins can co-assemble and be trafficked to the vacuole, where they are presumably destroyed.

2. Please amend the Materials and Methods to include the requested information.

We made the requested changes, in addition to a few others made to improve clarity.

3. Please mend the indicated figures to include MW information in figures S5D and S6C.

We added the MW labels to all Westerns.

4. I downloaded the original files for figures S5 and S6 and I think the quality of the figures is acceptable (and the other reviewers did not express any concern) - responding to this concern is not necessary.

5. I see what the reviewer is referring to, but I don't know enough about pombe morphology to know if it is a problem. At a minimum, please comment on this in your response letter. If you think there is an underlying issue that would alter interpretation of the experiments a more robust response may be warranted.

We do see variation in cell length in our experiments depending on the growth of the cultures. Those that saturate sooner tend to end up with shorter cells. The phenotype isn’t repeatedly observed with the genotypes shown and does not affect the relative timing of the Wtf^antidote^ signals (e.g. Figure 1C-F and S2 Figure B-C). Below is another image (not in the paper) of the same strains with more similar cell lengths, but the same earlier expression of Wtf4^antidote^-mCherry than mCherry-Wtf4^antidote^.

**Comments to the Authors:
Please note here if the review is uploaded as an attachment.**

Reviewer #1: This is a revised version of a manuscript previously submitted to PLoS Genetics. I can appreciate the efforts that the authors have done to improve the manuscript, following the comments from the reviewers and the suggestions from the editor, including adding new figures and entirely re-writing the manuscript.
Regarding my previous concerns, I am satisfied with the replies from the authors and with the specific changes that they have introduced in the manuscript, with one exception that I believe that should be corrected to improve the manuscript. I have serious concerns with the WB that they have included in the supplementary information (Figures S5 and S6):

- I understand that all the meiosis in the manuscript are in a “wild type” background, and that could be a reason for not using an “artificial” pat1-driven meiosis. But in view of the WB that the authors are showing, I think that they should use a pat1-ts background for the only WB experiments; pat1-driven meiosis allows a highly synchronous meiosis (much better than a h+/h- diploid meiosis) and might allow to detect some of the tagged proteins that so far they are not detecting (i.e. antidote-mCherry, Fig S5; mCherry-Antidote and Antidote-mCherry, Fig S6).
As detailed above, we have now included Western blots from *pat1* mutant meiosis.

There may be a band corresponding to Antidote-mCherry in the new blot, but it is not

robust. The signal is still mostly from free mCherry. This is likely because the antidote

signal we see in cells is in the vacuole, where the full-length protein is likely quickly

degraded. Given our failure to definitively observe full-length protein from Antidote-

mCherry (which has a stronger signal; S6 Figure) in *pat1* mutant meiosis, we did not

attempt similar mCherry-Antidote experiments in that background.

- What kind of protein extracts are the authors preparing? It is not indicated in the Materials and Methods section, neither the buffer in which cells are broken. If the authors suspect a problem with stability of the proteins (as it happens with many proteins expressed during meiosis), they should prepare TCA extracts; if there is a problem of solubility, then boiled extracts (running membrane fraction in the electrophoresis).

We have now added to the materials and methods to provide this important information. We did originally try TCA extracts, but still observed mostly free tags and no full-length proteins. We also tried boiled extracts. This, in addition to several other things we tried, was also unsuccessful.

- Molecular weight markers are not indicated in Fig S5D and S6C; in Figures S5BC and S6B, only 4 markers are indicated.

We added these labels to all the Western blots.

- The quality of fluorescence in these two figures is poor, compared to other figures in the same manuscript.

- Fig S6: why cells are so different in size at T=0 hours in the two top panels, compared with the bottom panel or with other figures? Cells with the empty vector or with the mCh-antidote are elongated in this specific experiment, indicating that probably there is something wrong with these two cultures.

See response and image above. In addition, cultures that are sickly fail to make it to the

end of meiosis. The cultures in Fig S6 completed meiosis with the expected efficiency, so we did not consider them compromised.

Reviewer #2: In their revised article on S. pombe wtf meiotic drivers, Nuckolls et al. investigate the wtf4 spore killer. The authors have satisfactorily addressed all of my questions concerning their initial version of the manuscript. I believe the current version of the manuscript represents a significant and valuable contribution to our understanding of spore killer-based gene drivers.

Minor suggestions:
Line 120 - maybe "diver" should be "driver"
Line 187 - maybe delete the first occurrence of "that"
Line 199 - maybe change to "…lacks introns, and thus…"
Line 230 - maybe delete "from at timepoints" and "in timepoints"

We made the suggested changes.

Reviewer #3: The manuscript is greatly improved and many of my comments have been addressed.
